# Contemporary evolution of resistance at the major insecticide target site gene *Ace-1* by mutation and copy number variation in the malaria mosquito *Anopheles gambiae*

**DOI:** 10.1111/mec.13197

**Published:** 2015-05-14

**Authors:** David Weetman, Sara N Mitchell, Craig S Wilding, Daniel P Birks, Alexander E Yawson, John Essandoh, Henry D Mawejje, Luc S Djogbenou, Keith Steen, Emily J Rippon, Christopher S Clarkson, Stuart G Field, Daniel J Rigden, Martin J Donnelly

**Affiliations:** * Department of Vector Biology, Liverpool School of Tropical MedicinePembroke Place, Liverpool, UK; † Department of Immunology and Infectious Diseases, Harvard School of Public HealthBoston, MA, USA; ‡ School of Natural Sciences and Psychology, Liverpool John Moores UniversityLiverpool, UK; § Biotechnology and Nuclear Agriculture Research Institute, Ghana Atomic Energy CommissionKwabenya, Accra, Ghana; ¶ Department of Molecular Biology and Biotechnology, University of Cape CoastCape Coast, Ghana; ** Department of Wildlife and Entomology, University of Cape CoastCape Coast, Ghana; †† Infectious Diseases Research CollaborationKampala, Uganda; ‡‡ Institut Regional de Sante Publique de OuidahOuidah, Benin; §§ Universite d'Abomey-CalaviCotonou, Benin; ¶¶ Department of Microbiology, Immunology & Pathology, Colorado State UniversityFort Collins, CO, USA; *** Institute of Integrative Biology, University of LiverpoolLiverpool, UK; ††† Malaria Programme, Wellcome Trust Sanger InstituteHinxton, Cambridge, UK

**Keywords:** *Ace-1* G119S, Acetylcholinesterase, gene duplication, malaria mosquito, purifying selection

## Abstract

Functionally constrained genes are ideal insecticide targets because disruption is often fatal, and resistance mutations are typically costly. Synaptic acetylcholinesterase (AChE) is an essential neurotransmission enzyme targeted by insecticides used increasingly in malaria control. In *Anopheles* and *Culex* mosquitoes, a glycine–serine substitution at codon 119 of the *Ace-1* gene confers both resistance and fitness costs, especially for 119S/S homozygotes. G119S in *Anopheles gambiae* from Accra (Ghana) is strongly associated with resistance, and, despite expectations of cost, resistant 119S alleles are increasing significantly in frequency. Sequencing of Accra females detected only a single *Ace-1* 119S haplotype, whereas 119G diversity was high overall but very low at non-synonymous sites, evidence of strong purifying selection driven by functional constraint. Flanking microsatellites showed reduced diversity, elevated linkage disequilibrium and high differentiation of 119S, relative to 119G homozygotes across up to two megabases of the genome. Yet these signals of selection were inconsistent and sometimes weak tens of kilobases from *Ace-1*. This unexpected finding is attributable to apparently ubiquitous amplification of 119S alleles as part of a large copy number variant (CNV) far exceeding the size of the *Ace-1* gene, whereas 119G alleles were unduplicated. *Ace-1* CNV was detectable in archived samples collected when the 119S allele was rare in Ghana. Multicopy amplification of resistant alleles has not been observed previously and is likely to underpin the recent increase in 119S frequency. The large CNV compromised localization of the strong selective sweep around *Ace-1*, emphasizing the need to integrate CNV analysis into genome scans for selection.

## Introduction

Detection of the genomic signals created by selective sweeps is a major goal of applied evolutionary studies aiming to discover variants associated with medically relevant phenotypes (Nair *et al*. 2003; Hedrick 2013; Karlsson *et al*. 2014). Yet this objective may be challenging in large populations because of their inherent capacity to generate and harbour mutations (Barton 2010; Karasov *et al*. 2010). This increases the likelihood of adaptation from standing genetic variation, leaving much weaker ‘soft sweep’ genomic signatures of selection than classical ‘hard sweeps’ from *de novo* mutation (Messer & Petrov 2013). Though less well investigated, signals of selection may be further obscured by structural complexities of genomes, such as (eu- vs. hetero-) chromatin variation leading to highly variable background recombination rates, polymorphic inversions and copy number variants (CNVs).

Mosquitoes typically exhibit many features that could create difficulties for the detection of selective sweeps in their genomes, including the following: large census and effective population sizes (Lehmann *et al*. 1998; Touré *et al*. 1998); extremely high genetic diversity and associated lack of linkage disequilibrium (LD) (Wilding *et al*. 2009; Neafsey *et al*. 2010; Weetman *et al*. 2010); many polymorphic paracentric inversions (Coluzzi *et al*. 2002; Pombi *et al*. 2008); and chromatin-type-linked recombination rate variation spanning orders of magnitude within the same chromosome (Pombi *et al*. 2006). The prevalence and general importance of CNVs in mosquito genomes remains to be quantified. Recent work on *Drosophila* spp. (Rogers *et al*. 2014) suggests that CNVs may regularly fuel *de novo* adaptation, and their evolutionary significance in mosquitoes might well be underappreciated.

In this study, we focus on the magnitude and nature of genomic signals of selection within and around the *Ace-1* gene, which encodes synaptic AChE in mosquitoes. Acetylcholinesterase hydrolyses the neurotransmitter acetylcholine to terminate nerve signal transmission in synapses and is one of only two target sites for the major chemical insecticide classes currently available for malaria vector control. Carbamate and organophosphate insecticides both bind to and inhibit AChE, which results in accumulation of acetylcholine in the nerve synapse, leading to paralysis and eventual death of the insect. Owing to widespread resistance to DDT and pyrethroids in the major *Anopheles* malaria vectors (Ranson *et al*. 2011), carbamates and organophosphates are being used increasingly for vector control across sub-Saharan Africa (Sharp *et al*. 2007; Akogbéto *et al*. 2010; Kigozi *et al*. 2012). Whether this increased use for vector control is selecting for enhanced resistance is unclear, but in general, *Ace-1* variants associated with resistance are expected to be primarily selected by exposure to insecticides, which have only been available for a maximum of 60 years. Only three amino acid substitutions in *Ace-1* have been associated with insecticide resistance in mosquitoes (Alout & Weill 2008), and only one of these, G119S (using *Torpedo californica* codon nomenclature), has been found in *Anopheles*. In *A. gambiae* and its sibling species *Anopheles coluzzii* (formerly known as the *A. gambiae* s.s. S and M molecular forms), resistance to carbamates and organophosphates conferred by *Ace-1* 119S is currently restricted to West Africa (Ahoua Alou *et al*. 2010; Essandoh *et al*. 2013). G119S is located close to the catalytic site of AChE, and 119S-bearing AChE has very similar biochemical properties in *Culex* and *Anopheles* (Alout & Weill 2008). This suggests that not only will 119S-generated resistance profiles to carbamate and organophosphate be similar in each species (Alout *et al*. 2008), but also that the multiple deleterious effects of 119S documented in *Culex* spp. (especially for homozygotes) will also apply in *Anopheles* (Djogbénou *et al*. 2010).

Partial sequencing of *Ace-1* has detected only a single haplotypic background for the 119S allele in both *A. gambiae* and *A. coluzzii*, consistent with a single origin (Djogbénou *et al*. 2008b; Essandoh *et al*. 2013), possible introgression between the species and subsequent spread via a hard selective sweep. However, mutational options within functionally critical, evolutionarily conserved genes such as *Ace-1* are likely to be limited (Weill *et al*. 2003; Oakeshott *et al*. 2005; Remnant *et al*. 2013), which might lead to conflation or confusion of the reduced diversity expected from purifying and directional selection, and possible overestimation of the latter. Duplication of *Ace* genes is well known in agricultural pests (Bass & Field 2011) and, in the best understood example of a CNV of contemporary importance in mosquitoes, *Ace-1* duplicants are positively selected in insecticide-exposed field populations of *Culex pipiens* (Labbé *et al*. 2007b). Here, creation of a permanent ‘heterozygote’ from phased pairing of 119G and 119S alleles (Labbé *et al*. 2007a) compensates for the compromised neurophysiological performance of 119S in the absence of insecticide (Alout *et al*. 2008). *Ace-1* duplication has also been found in both *A. gambiae* and *A. coluzzii* (Djogbénou *et al*. 2008a; Essandoh *et al*. 2013) and, as in *C. pipiens*, only in G119S heterozygotes. In a multiple-insecticide resistant population of *A. coluzzii* from Côte d'Ivoire in which almost all individuals type as G119S heterozygotes and 119S/S homozygotes are never found (Ahoua Alou *et al*. 2010; Edi *et al*. 2012), possession of duplicated resistant alleles is very strongly associated with survival following carbamate exposure in bioassays (Edi *et al*. 2014).

In contrast to the most important mutation (*Vgsc* ‘*kdr*’ 1014F) in the only other major insecticide target site, the voltage-gated sodium channel (VGSC), for which a very strong signature of a hard selective sweep has been detected in field populations (Lynd *et al*. 2010), the extent of selection on *Ace-1* mutation in *Anopheles* natural populations is unknown. Even with increasing use of *Ace-1*-targeting insecticides, the net outcome of selection on *Ace-1* in the field is difficult to predict because of the expectation of strong fitness costs for the resistant serine allele. Here, we apply comparative sequencing, genotyping and qPCR to *A. gambiae* samples, chosen for homozygosity at the G119S position to facilitate detection of genomic differentiation, from a location of high-prevalence carbamate and organophosphate resistance in southern Ghana. Specifically, we aimed to investigate the following: (i) whether there is significant genomic evidence of selection, and its nature, within and around the *Ace-1* gene; (ii) how temporal variation in 119S frequency might correspond with signatures of selection; (iii) whether a simple hard selective sweep model could explain any detectable signals of selection.

## Materials and methods

### Samples and diagnostic SNP genotyping

Mosquito larval collections were performed using the standard dipping method in May 2008 from Dzorwulu, Madina, Labadi and Roman Ridge, suburban locales of Accra (5.55°N, 0.20°W) in southern Ghana, and in May 2010 from Madina. Larvae were reared at the Biotechnology and Nuclear Agriculture Research Institute, Accra. Pupae were picked daily and placed into plastic cages. Emergent *A. gambiae s.l*. females were maintained on 10% sugar solution until 3–5 days posteclosion when insecticide bioassays were performed. Mosquitoes were exposed to 0.1% bendiocarb for one hour following the WHO tube assay protocol (WHO 2013). Mortality was assessed 24 h after the end of the exposure period, and mosquitoes were preserved individually over silica gel. Collections of additional female specimens from 2002 (several locations within the Greater Accra district), 2007 (Madina) and 2011 (Madina) followed similar protocols, and the collection sites are detailed elsewhere (Yawson *et al*. 2004; Lynd *et al*. 2010; Essandoh *et al*. 2013).

DNA was extracted using a DNEasy Blood & Tissue kit (Qiagen). Two standard methods were employed for molecular species identification of the morphologically identical species within the *A. gambiae* s.l. complex. The first involves PCR amplification of IGS rDNA towards the centromere of the X chromosome using a cocktail of *A. gambiae* complex species-specific primers, followed by restriction digest of products and visual diagnosis of species-diagnostic fragments on agarose gel (Fanello *et al*. 2002). The second, which exploits a species-specific SINE insertion polymorphism to generate diagnostic fragments of different size (Santolamazza *et al*. 2008), discriminates among fewer species in the complex but is particularly reliable for distinguishing between the sibling species pair *A. gambiae* and *A. coluzzii*. Both methods provided entirely congruent results. All samples were further characterized for their genotype at the *Ace-1* G119S polymorphism using a standard TaqMan quantitative PCR assay (Bass *et al*. 2010).

### *Ace-1* gene sequencing and analysis

Twenty-five female *A. gambiae* from the 2008 collection that were homozygous for the *Ace-1* G119S polymorphism were chosen for sequencing of a region of the *Ace-1* gene (VectorBase gene ID: AGAP001356). Eleven individuals were wild-type (119G) homozygotes, and 14 were resistant (119S) homozygotes. We used an existing published protocol (Djogbénou *et al*. 2008b) for initial amplification of a fragment including the 119 codon using the primers AgEx2dir1 (5′-AGGTCACGGTGAGTCCGTACGA-3′) and AgEx4rev2 (5′-AGGGCGGACAGCAGATGCAGCGA-3′). PCRs contained 2.5 μL of 10× Bioline buffer, 0.2 μm of each dNTP, 0.2 μm of each primer, 0.75 U BioTaq (Bioline), 1 μL genomic DNA template and water to a total volume of 25 μL. PCR conditions were as follows: 94°C for 3 min, followed by 35 cycles of 94 °C for 30 s, 56 °C for 30 s and 72 °C for 60 s, with a final extension at 72 °C for 5 min. Products were cleaned using QIAquick PCR Purification kits and sent to Macrogen (Korea) for bidirectional sequencing using the same primers as for product amplification. Additional primers (Ace1_5F: 5′-GATCGGAGAACAGGCATCAT-3′ and Ace1_5R: 5′-CCACTTCCAATCGCGTACTT-3′; Ace1_3F: 5′-AGGTGCTCTTCTTCCCATCA-3′ and Ace1_3R: 5′-CTCGGTCCAGTCGGTGTACT-3′) were designed to provide a total of approximately 2000 bp of sequence around the 119 codon in 5′ and 3′ directions. PCR amplicons were generated using the same conditions as above, except for the addition of 0.5 μL 50 mm MgCl_2_ to the reaction mixture and the use of a slightly higher annealing temperature (57 °C). The additional amplicons were cleaned and sequenced as before. If intronic indels resulted in poor quality direct sequence data, or where it was impossible to computationally phase the data due to high sequence polymorphism (Stephens *et al*. 2001), PCR amplicons were cloned using a pGEM T-Easy Vector (Promega), according to the manufacturer's instructions, and then sequenced as before. Contigs were aligned and edited in CodonCode aligner v4 (CodonCode Corporation) and annotated in DnaSP v5 (Librado & Rozas 2009).

### *Ace-1* sequence analysis

Extended haplotype homozygosity (EHH) analysis (Sabeti *et al*. 2002) was carried out to assess the patterns of linkage disequilibrium associated with wild-type (119G) and resistant (119S) alleles. As in a previous study of linkage disequilibrium (LD) decay around the *Vgsc* 1014F *kdr* mutation (Lynd *et al*. 2010), we defined the core of the haplotypes as the non-synonymous mutation at codon 119 and then examined the decay in LD in both telomeric and centromeric directions in both wild-type and resistant haplotypes. Identification of polymorphic sites within the data sets and subsequent EHH analysis was conducted using programs written in R (R Development Core Team 2014). Significant differences in EHH values were determined via non-overlapping 95% confidence intervals (CI), calculated at each SNP position using a bootstrapping procedure, with 1000 resamples. An illustrative bifurcation plot was produced in SWEEP v2.1.1 (Sabeti *et al*. 2002). To investigate evidence for purifying selection acting on the *Ace-1* locus, the sequence haplotypes bearing the wild-type 119G variant were subject to Tajima's D and Fu and Li's D and F tests in DnaSP v5 (Librado & Rozas 2009). Haplotype trees were constructed separately for non-synonymous and synonymous mutations using the statistical parsimony algorithm in TCS 1.21 (Clement *et al*. 2000).

### Modelling the impact of non-synonymous substitutions on AChE function

*In silico* modelling was used to investigate whether non-synonymous changes (other than G119S) observed in the sequenced haplotypes might impact on the catalytic capacity of the AChE enzyme and its ability to withstand carbamate inhibition. A molecular model of *A. gambiae* AChE was constructed with the aid of the HHpred server (Söding *et al*. 2005) using the well-characterized enzyme from *Torpedo californica* (Colletier *et al*. 2006) as a single top-scoring template. The target and template sequences share 48% sequence identity that, along with the existence of only six short (1–3 residue) indels, ensures a reliable model in the catalytic domain. N- and C-terminal extensions in the insect target sequence of around 160 and 40 residues, respectively, did not align with the template and so could not be modelled. Using PyMOL (https://www.pymol.org), the position of each observed amino acid change was visualized relative to the catalytic and accessory substrate binding sites (Colletier *et al*. 2006). The local structural context derived from the model was used to predict any likely impact on protein structure of the mutations. An alignment of orthologous insect AChE enzymes, made with MAFFT (Katoh & Standley 2013), applied to homologues obtained from the HHpred server, was used to determine residue conservation and whether the allele residues were seen at corresponding positions in other AChE enzymes.

### Microsatellite screening around *Ace-1* to define the extent of the hitchhiked region

Microsatellites were identified in a region of 2 Mb either side of the *Ace-1* locus from the *A. gambiae* PEST AGAMP3 genome assembly (Holt *et al*. 2002) using SciRoKo (Kofler *et al*. 2007). Twenty-four di- and tri-nucleotide loci, approximately symmetrically distributed about the *Ace-1* G119S position, and at progressively increasing distances between neighbouring loci (further from *Ace-1*), were selected for screening. All exhibited a minimum of eight uninterrupted repeats in the PEST genome. Primers were designed to amplify products 100–300 bp using Primer3 (Koressaar & Remm 2007; Untergasser *et al*. 2012) (Table S1, Supporting information). Loci were amplified individually using 2.5 μL of 10× Kapa Taq buffer (Kapa Biosystems), 0.2 μm of each dNTP, 0.2 μm of each primer, 1 U KapaTaq, 2.5 μL 2 mm MgCl_2_, 1 μL genomic DNA template and water to a total volume of 25 μL. PCR conditions were as follows: 95°C for 3 min, followed by 30 cycles of 95 °C for 30 s, *n* °C for 30 s (see Table S1, Supporting information, for locus-specific values of *n*) and 72 °C for 30 s, with a final extension at 72 °C for 5 min and then pooled in subsets of four or five (differentiated by product size) for genotyping on a CEQ 8000 capillary sequencer (Beckman-Coulter), with cy5 or cy5.5 5′-labelled forward primers and a 400-bp-size ladder (Beckman-Coulter). All samples with *Ace-1* sequence data were genotyped at the microsatellite loci along with a further 45 *A. gambiae* homozygotes (119S/119S or 119G/119G) from the same sample collection. Samples were randomized on plates for genotyping, and any failed microsatellite PCR was repeated at least once. All microsatellite scoring of automatically sized alleles was performed by a single experienced operative.

### Microsatellite data analysis

Micro-Checker 2.2.3 (Van Oosterhout *et al*. 2004) was used to examine genotype data for null alleles and possible scoring errors arising from PCR dropout of large alleles and mis-scoring of stutter peaks as alleles (or *vice versa*). Where identified, scores were rechecked and specific samples regenotyped as necessary to help clarify scoring. Standard population genetic metrics: *F*-statistics, heterozygosity and tests for (genotypic) population differentiation were calculated using FSTAT 2.9.1 (Goudet 1995). F_ST_ was adjusted for downward bias arising from locus heterozygosity (Hedrick 2005), and expressed as *F*_ST_’, using RecodeData v 0.1 (Meirmans 2006). Tests for Hardy–Weinberg and linkage equilibrium were performed using GENEPOP 4.2 with default settings (Rousset 2008). All final-call microsatellite genotyping scores are provided in file Data S1 (Supplementary data set).

To check scoring of a specific microsatellite locus ‘Ace-5k’ (within an intron of the *Ace-1* gene), we sequenced several 119G/G and 119S/S samples. Amplification of a 547-bp fragment proceeded using reaction mixtures contained 2.5 μL of 10× Fermentas Dream buffer, 0.2 μm of each dNTP, 0.2 μm of each primer (min5ace_bF: 5′-GCATCGCGGGAAACATTTTG-3′; min5ace_bR: 5′-CGCTTTGCAGTGTTGTCCTT-3′), 0.5 U Fermentas Dream Taq, 1 μL genomic DNA template and water to a total volume of 25 μL. PCR conditions were as follows: 94 °C for 3 min, followed by 35 cycles of 94 °C for 30 s, 54 °C for 30 s and 72 °C for 30 s, with a final extension at 72 °C for 5 min. Products were cleaned using a QIAquick PCR Purification kit and sent to Macrogen (Korea) for bidirectional sequencing using the same primers as for product amplification.

### Inference of a selective sweep from patterns of diversity across microsatellites

For a population at mutation–drift equilibrium, the expected heterozygosity (H_E_) at a microsatellite locus can be used to estimate the parameter θ = 4*N*eμ, where *N*e is the effective population size and μ is the mutation rate. By comparing locus-specific estimates of θ between populations, or in this instance, groups defined by their genotypes at the *Ace-1* codon, it is possible to perform a diversity-based outlier analysis to identify loci which may have been subject to selection (Harr *et al*. 2002). The ratio of θ estimates is calculated from the ratio of expected heterozygosity (H_R_/H_S_*,* where H_R_ here is heterozygosity in 119S/119S individuals and H_S_ is heterozygosity in 119G/119G individuals). When natural log-transformed H_R_/H_S_ conforms to a normal distribution over a range of demographic scenarios (Schlötterer 2002), therefore, the normal probability density function can be used to ascribe significance to estimates for each locus (Harr *et al*. 2002). Unbiased estimates of heterozygosity with bootstrapped 95% CI for the ln(H_R_/H_S_) statistics were calculated using scripts written in R (R Development Core Team 2014). The specimens sequenced were all collected in May 2008 from Accra but encompassed several local suburbs. In case grouping of spatial samples impacted diversity estimates, the analysis was repeated upon a subset of approximately 40% of the specimens from a single suburb, Dzorwulu.

We modelled whether the pattern of reduced variability around the *Ace-1* locus was concordant with our estimate of selection derived from temporal series data (2007, 2008, 2010 and 2011 samples). We used the approach described for studies of pyrimethamine resistance in *Plasmodium falciparum* (Nair *et al*. 2003). The expected reduction in heterozygosity in *Ace-1* 119S homozygotes E(H_R_) was inferred based on the observed heterozygosity at each locus in wild-type susceptible *Ace-1* 119G homozygotes (H_S_) using the following equation (Wiehe 1998; Nair *et al*. 2003):


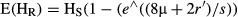


A broad-scale chromosome arm 2R-specific recombination rate (*r*’) of 1.33 cm/Mb was calculated from published mapping data (Zheng *et al*. 1996) (Fig. S1, Supporting information). An estimate of the selection coefficient (*s*) was obtained from the temporal G119S frequency data using a simple codominant model of bi-allelic selection in the software populus, with predictions from a linear regression used to provide initial and test values. The value of *s* (to three decimal places) best approximating the data was determined as that minimizing a chi-square goodness-of-fit statistic in comparisons of test values to simulations. A species-specific microsatellite mutation rate estimate of μ = 3.4 × 10^−5^ was used (Lehmann *et al*. 1998).

To determine the fit of observed H_R_ values to the E(H_R_) predictions at individual loci, we applied an outlier analysis based on the absolute difference between the predicted value for each locus and, to allow for observational uncertainty, the appropriate confidence limit (i.e. whichever is closer to prediction) of the observed data. Outlier analyses proceeded by calculating the median of the absolute deviation of these abs(CI obs − exp) values from their median, and multiplying by a constant (*b* = 1.4826) representing the normal distribution to yield a median absolute deviation metric (*MAD*) (Leys *et al*. 2013). Outliers were considered as data points whose value was more extreme than 3× *MAD*, which represents a conservative threshold (Leys *et al*. 2013).

### *Ace-1* copy number variation screening

All microsatellite-screened *A. gambiae* were also subject to a quantitative PCR diagnostic to identify and quantify *Ace-1* genomic copy number variation (Edi *et al*. 2014). In brief, three fragments from different exons of the *Ace-1* gene are amplified and normalized to two single copy genes (*Cyp4g16* and *elongation factor Tu*). Copy number of *Ace-1* was estimated relative to two pools of gDNA from females of the standard multi-insecticide susceptible Kisumu laboratory strain (established in 1975) using the ΔΔCT method (Schmittgen & Livak 2008). Pools provide equivalent estimates to single individuals and were used simply to provide a longer-lasting source of reference DNA. Copy number estimates are represented as mean ΔΔCT with standard deviations to indicate ΔΔCT variability across the three *Ace-1* fragments. To ensure cross-comparability of estimates from the 2008 and 2002 samples (performed in separate batches), we included *Ace-1* 119 G/G and 119 S/S homozygotes that served as controls for unduplicated and duplicated genotypes, respectively. Consistency of the three ΔΔCT estimates was assessed from their standard deviation, which were investigated for outliers using the same *MAD* method as described above. Two outlying samples were identified as having exceptionally high standard deviations and removed from the 2008 sample data set; no outliers were present in the 2002 data (Data S1, Supplementary data set). Tests for differences in mean estimates between sample groups were performed using either homoscedastic or heteroscedastic two-tailed *t*-tests depending on the result of Levene's F-test for homogeneity of variances. Spearman rank correlations, Levene's test, *t*-tests and Wilcoxon signed-rank tests were conducted in SPSS v20.

## Results

### *Ace-1* sequence analysis

**Fig. 1:**
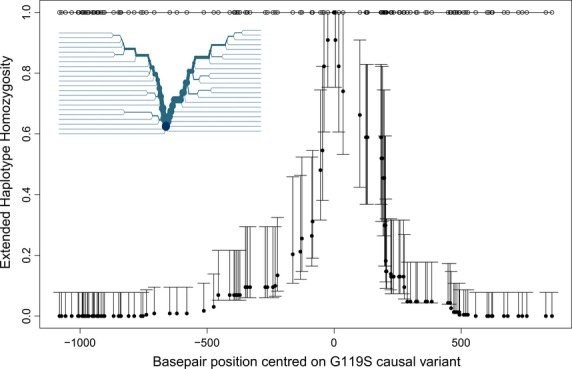
EHH analysis showing LD decay with increasing distance from the 119 codon core position (marked as the origin on the *x*-axis) in *Anopheles gambiae* from Accra. Negative numbers are SNPs in the telomeric (T) direction and positive numbers in the centromeric (C) direction plotted on a physical scale with a total span of approximately 2000 bp. For the 119G wild-type data (filled circles), the 95% CI were estimated by bootstrapping. Only a single haplotype was associated with the 119S resistance mutation (open circles), and therefore, EHH = 1 for all SNP positions. Inset panel: long-range haplotype bifurcation plot for 119G (119S shows no bifurcation) illustrating patterns of recombination in each direction, with orientation as in main panel. The core is marked by the dark circle; each SNP is represented by a node; and a recombination (or possibly mutation) event is represented by a bifurcation. The diameter of the circle at each SNP node is proportional to the number of individuals with the same long-range haplotype at that position.

Sequences were analysed from *A. gambiae* individuals collected in 2008 from Madina and other suburbs of Accra. A total of 124 SNPs, and a number of indels in intronic regions, were identified in 2031 bp of sequence from within *Ace-1*. Consistent with a strong, allele-specific selective sweep, the (resistant) 119S mutation occurred on a single haplotypic background, whereas extended haplotype homozygosity (EHH) decayed very rapidly in both telomeric and centromeric directions for 119G (i.e. wild type, susceptible) haplotypes (Fig.1), all of which required cloning and sequencing for resolution owing to very high diversity within individual sequences.

Analysis of 119G haplotypes revealed a marked skew in the ratio of synonymous to non-synonymous mutations (46:8 in the 1266 bp of exonic sequence examined, comprising of approximately 60% of the coding region of *Ace-1*), consistent with the action of purifying selection. This was supported by Fu and Li's tests of selection (D* = −2.65, *P* < 0.05; F* = −2.73, *P* < 0.05), with Tajima's test bordering significance (*D* = −1.62, 0.10 > *P* > 0.05). Contrasting statistical parsimony networks constructed from non-synonymous vs. synonymous variants provide further support to the hypothesis of strong purifying selection. The non-synonymous network shows a starlike pattern of short branches (most one substitution) radiating from the most frequent haplotype, which is presumably the ancestral wild type (Fig. S2A, Supporting information). The synonymous network exhibits substantial accumulation of mutations within often long branches separating manifold lineages, which are interconnected by extensive reticulations indicative of many recombination events (Fig. S2B, Supporting information).

Each of the eight non-synonymous substitutions detected was observed in only a single haplotype, although one of these haplotypes contained three changes (Fig. S2A, Supporting information). Structural modelling suggested that, of the seven variant amino acid positions that could be modelled (Table S2, Supporting information), six are conservative and positioned too far from the catalytic and secondary binding sites to radically influence substrate binding. The final substitution is relatively close to the catalytic site but exhibits many variant amino acids in close orthologues, suggesting that polymorphism at this codon is unlikely to seriously impact AChE function. Thus, the few rare non-synonymous variants detected in the 119G haplotypes seem likely to be of limited functional significance.

### Resistance association and temporal variation of *Ace-1* 119S

A total of 561 female *A. gambiae* s.l. (*A. gambiae* and *A. coluzzii*) collected from several Accra suburbs in 2008 were exposed to bendiocarb in standard tube bioassays. A further sample of 192 ‘controls’ were preserved without insecticide exposure. Of these, 333 females – either survivors of bendiocarb exposure or controls – were identified to species using PCR diagnostics and genotyped at the *Ace-1* 119 locus. Although our study focuses on *A. gambiae*, we also include *A. coluzzii* results here for comparison. *Ace-1* 119S was found at significantly higher frequency in bendiocarb assay survivors than in the unexposed controls in both *A. gambiae* and *A. coluzzii* (Table 1A). Based on the proportions of *A. gambiae* and *A. coluzzii* in the subset of the total bioassay sample that were diagnosed to species, the overall estimated mortalities differed dramatically (*A. gambiae* = 0.39, 95% CL 0.30–0.43; *A. coluzzii* = 0.99 95% CL 0.96–0.99).

**Table 1:** Association of G119S with bendiocarb resistance in females from Accra, Ghana

	*Anopheles gambiae*		*Anopheles coluzzii*	
	Alive	Pop freq	Alive	Pop freq
(A) 2008 Genotype				
119G/G	0	33	1	94
119G/S	88	30	3	24
119S/S	48	10	1	1
Total N	136	73	5	119
Probability	5.5 × 10^−11^		0.004	

	*A. gambiae*		*A. coluzzii*	
	Alive	Dead	Alive	Dead
(B) 2010 Genotype				
119G/G	0	15	0	8
119G/S	25	2	1	1
119S/S	2	0	0	0
Total N	27	17	1	9
OR (95% CL)	18.6	(4.0–85.3)	17	(0.5–523.8)
Probability	7.6 × 10^−6^		0.19	

(A) Frequency of each *Ace-1* G119S genotype in the insecticide-unexposed Accra population (mixed suburbs as in sequence data) and in bioassay survivors from the same collection sites for *A. gambiae* and *A. coluzzii*. (B) Genotypes of females from the Madina suburb of Accra, alive and dead following bendiocarb bioassays. Footnotes show probability of (no) association from Fisher's exact tests and (in B) the allelic odds ratio (OR) for association of G119S with bioassay survivorship.

An altered protocol in the 2010 sample collection from Madina (Accra) permitted both the surviving and dead mosquitoes from bendiocarb bioassays to be genotyped, which yielded a more direct demonstration of the very strong association of the *Ace-1* G119S substitution with resistance (Table 1B). In both the 2008 and 2010 collections, *A. coluzzii* showed limited bendiocarb resistance and low frequencies of 119S alleles, in agreement with a broader scale study across southern Ghana (Essandoh *et al*. 2013).

**Fig. 2:**
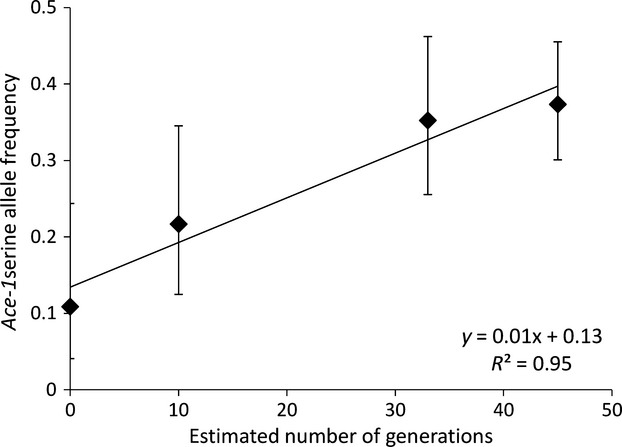
Frequency (with 95% CI) of the *Ace-1* 119S-resistant allele in *Anopheles gambiae* from the suburb of Madina in Accra over a period of approximately 4 years. The linear trend represented by the regression is highly significant (Cochrane–Armitage 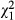 = 14.2, *P* = 0.0002), whereas the nonlinear component is not (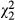 = 0.88, *P* = 0.64).

We added samples of *A. gambiae* collected in Madina in 2007 and 2011 to those from 2010 and the Madina portion of the 2008 collection, to investigate temporal variation in 119S frequency, without potential for spatial confounding. Data were fitted well by a linear model, assuming 12 generations per year (Fig.2), and genotypes in each collection conformed to Hardy–Weinberg expectations (minimum *P* = 0.23). The frequency of the resistant 119S allele increased strongly during the sampling period (Fig.2). From a codominant model, which appears appropriate based on the observed linearity and correspondence of data to Hardy–Weinberg proportions, we estimated a best-fit selection coefficient of *s* = 0.064 acting upon the resistant allele (Fig. S3, Supporting information).

### Wider selective sweep around *Ace-1* G119S

**Fig. 3:**
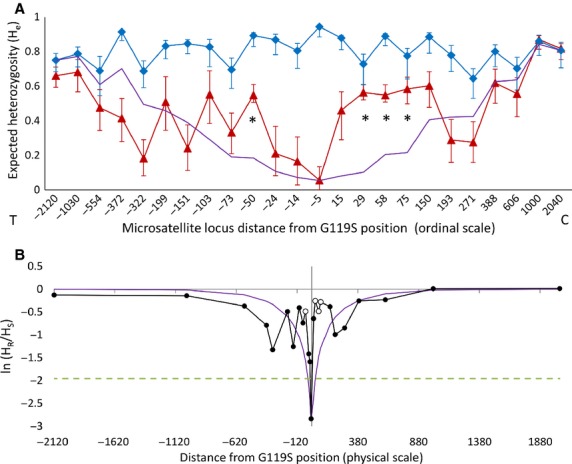
Variation in diversity at microsatellites distributed approximately symmetrically over 2 Mb around *Ace-1* G119S in all 2008 Accra samples of *Anopheles gambiae*. (A) Heterozygosity ±95% CI for 119S (resistant) homozygotes (red triangles) and 119G (wild-type) homozygotes (blue diamonds) plotted against an ordinal scale, numbered from the *Ace-1* G119S position in a telomeric (T) to centromeric (C) orientation. The purple line without markers is a deterministic model prediction for a hard selective sweep calculated from wild-type heterozygosities and realistic parameter estimates for the selection coefficient, mutation and recombination rates. *microsatellites detected as significant outliers by their (poor) fit to model prediction (B) Ratio of expected heterozygosities at each locus between groups: ln(H_R_/H_S_) plotted against a physical scale. The dashed line shows the 95% significance threshold for a two-tailed *Z*-test. The purple line without markers shows the equivalent model prediction to that in A; loci identified as outliers in A are shown as unfilled points.

A series of microsatellites located at progressively increasing distances from *Ace-1* codon 119 (Table S1, Supporting information) were screened in the 119G/G and 119S/S individuals sequenced previously, along with additional homozygote females obtained from the same collection (total N genotyped: 119G/G = 33; 119S/S = 36). In line with the high polymorphism observed in the sequence data, heterozygosity (H_e_) in the 119G/G group was high throughout the approximately 4.2 Mb region screened, especially in the vicinity of *Ace-1*. Confidence intervals for H_e_ overlap between 119G/G and 119S/S groups beyond −372 kb in the telomeric direction and 388 kb in the centromeric direction, suggesting a broadly symmetrical span of reduced diversity covering at least 760 kb (Fig.3A). The ln(H_R_/H_S_) statistic (Fig.3B) clearly pinpointed the centre of the impacted region to the ‘Ace-5K’ microsatellite, located in an intron of *Ace-1* and the closest locus to G119S. As expected for a selective sweep around a single target, the correlation between ln(H_R_/H_S_) and distance of loci from the G119S position was significantly positive (ρ = 0.65, *N* = 24, *P* = 0.0006); i.e. the diversity increased with distance from G119S.

Yet, comparison of ln(H_R_/H_S_) between microsatellites paired approximately by distance on either side of G119S revealed significant asymmetry, with higher 119S/S sample microsatellite diversity in the centromeric direction (Wilcoxon signed-rank test, *Z* = 2.41, *P* = 0.012). Fit of 119S/S sample data to the hard sweep model also appeared heterogeneous, with significant outliers detected at three adjacent loci 29–75 kb from G119S in the centromeric direction and at 50 kb in the telomeric direction (Fig.3A), reflecting an inconsistent model fit within regions 10s–100s of kb from G119S (Fig.3B).

**Fig. 4:**
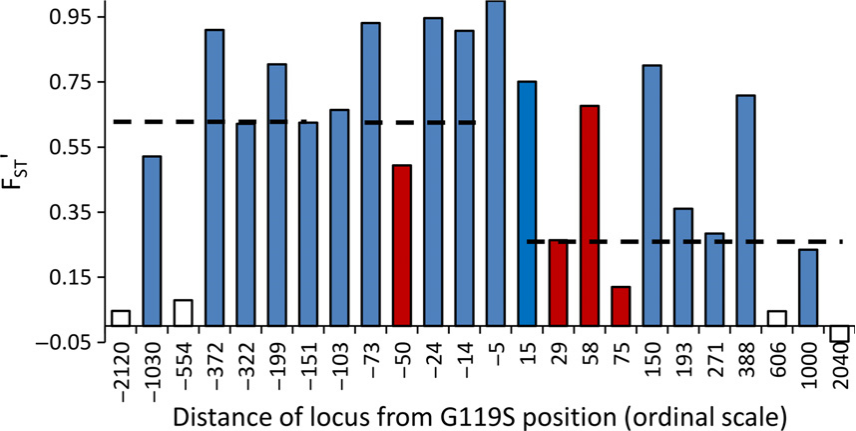
Microsatellite differentiation, measured as diversity-corrected *F*_ST_ between 119S/S (resistant) and 119G/G (susceptible) homozygote *Anopheles gambiae*. All loci showed significant differentiation (after correction for multiple testing) apart from some of the outermost loci, shown as unfilled bars. Dark red bars show model-fit outliers (see Fig.3). The medians for *F*_ST_’ across loci on each side of G119S are shown by dashed lines; loci at −103 and −5 are excluded for symmetry because there is no paired microsatellite location on the opposing side.

Differentiation between the G119S sample groups was generally high, most notably at the Ace-5K locus (*F*_ST_’ > 0.99), resulting from predominance of a single allele in the 119S/S samples that was absent from the relatively uniform frequency distribution of 25 alleles found in the 119G/G group (Fig. S4, Supporting information). Consistent with recombination between 119S and 119G chromosomes, differentiation was reduced towards the edges of the region screened, but significant *F*_ST_’ remained at loci approximately one megabase from G119S in each direction (Fig.4), suggesting potentially even wider bounds of the selective sweep than suggested from patterns of reduced diversity. However, asymmetry about *Ace-1* was again evident with significantly lower median *F*_ST_’ in the centromeric than in the telomeric direction (Wilcoxon signed-rank test *Z* = 2.13, *P* = 0.033).

### Evaluation of population genetic parameters suggests a large CNV

Patterns of microsatellite diversity and differentiation broadly correspond to expectations of a selective sweep centred on *Ace-1*, but with pronounced localized deviations from model predictions. We consider several possible explanations (numbered i-v below) for this relatively poor fit of the hard sweep model predictions.

A first possibility, that (i) admixture of samples from differentiated sampling locales might weaken signals of selection, was readily discounted. Repetition of ln(H_R_/H_S_) analysis for the subset of females from the single Accra suburb of Dzorwulu (*N* = 12 119S/S and *N* = 18 119G/G) provided near-identical results (Fig. S5, Supporting information) to those from the whole data set. Secondly, the ‘best estimate’ (ii) model parameter values applied might be inaccurate. Though this is certainly possible, when we repeated simulations allowing for substantial variation among the three key model parameters (within the ranges: recombination rate: 0.3–2.0; microsatellite mutation rate: 10^−4^–10^−6^; selection coefficient: 0.025–0.100), no parameter combination came any closer to replicating both the broad span of the sweep and the apparent rebound in diversity proximal to *Ace-1* (results not shown). A third explanation is the possible existence of heterogeneity in mutation rate among loci or, and perhaps more plausible given the short timescale over which selection has probably occurred, (iii) heterogeneity in recombination rate throughout the region genotyped. Recombination rate data are available for chromosome arm 2R on which *Ace-1* is located (*Ace-1* AGAMP4 position: 3 484 107–3 495 790; numbered from the telomere), but unfortunately, quantification accuracy is poor in the 4.2 Mb region genotyped here (Fig. S1, Supporting information). Nevertheless, variation in recombination rate might be most readily apparent via patterns of linkage disequilibrium (LD). As expected for a hard selective sweep, LD was significantly more pronounced among loci in the 119S/S group, but contrary to expectation, few of the significant LD tests were among microsatellite pairs close to *Ace-1* (Fig. S6, Supporting information). Whilst this recombination heterogeneity might be involved, such LD inconsistency could also have other causes, which we examined via patterns of Hardy–Weinberg deviation (measured by *F*_IS_).

Significant homozygote excess was detected at many loci, most likely a result of frequent (iv) null alleles (Table 2), which are a predictable consequence of the extreme SNP frequency in *Anopheles* and other mosquitoes (Wilding *et al*. 2009). Null alleles might weaken the selective sweep signal by inflating the ratio of H_R_/H_S_ (i.e. 119S/S H_E_: 119G/G H_E_) via reduced allelic diversity in 119G/G samples. However, null alleles in the 119S/S group would have the opposite effect and were almost as commonly detected as in the 119G/G samples. Crucially, though null alleles were suggested in 119G/G samples at three of the four loci showing especially poor model fit, a far more notable difference at these loci is that, in the 119S/S group, they are the only loci to exhibit a significant excess of heterozygotes (Table 2), arising from near-ubiquitous possession of the just two alleles (Fig. S4), Supporting information).

**Table 2:** Deviations from Hardy–Weinberg equilibrium (measured by *F*_IS_) and possible sources of scoring errors at microsatellite loci

	119S/S			119G/G		
Locus (kb)	*F*_IS_	*P*-value	Null freq	*F*_IS_	*P*-value	Null freq
−2120	−0.21	0.571		−0.17	0.050	
−1030	−0.01	0.348		−0.03	0.057	
−554	−0.16	1.000		0.22	0.066	
−372	0.00	0.513		0.09	0.427	
−322	−0.07	1.000		0.12	0.533	
−199	0.11	0.196		0.15	**0.000**	0.288
−151	0.05	0.445		−0.04	0.910	
−103	0.55	**0.000**	0.190	0.67	**0.000**	0.450
−73	−0.12	1.000		0.00	0.931	
−50*	−0.79	**0.000**		0.12	0.416	
−24	0.34	0.008	0.057	0.25	**0.000**	0.166
−14	0.65	**0.001**	0.090	0.63	**0.000**	0.273
−5	−0.01	1.000		0.46	**0.000**	0.215
15	0.64	**0.000**	0.198	0.12	0.036	
29*	−0.75	**0.000**		0.32	0.004	0.192
58*	−0.85	**0.000**		0.12	0.063	
75*	−0.33	**0.000**		0.36	**0.002**	0.374
150	0.54	**0.000**	0.199	0.36	**0.000**	0.301
193	−0.09	1.000		−0.01	0.021	
271	−0.11	1.000		0.18	0.488	
388	0.29	0.009	0.227	0.44	**0.000**	0.288
606	0.05	0.922		0.25	0.034	0.167
1000	−0.02	0.626		0.14	0.041	
2040	0.10	0.334		0.28	**0.001**	0.229

Loci are ordered by distance from *Ace-1* G119S with *F*_IS_ values and probabilities (for the null hypothesis of *F*_IS_ = 0), shown in bold where significant after Bonferroni correction for multiple testing. Null allele frequency estimates are shown where suggested as probable by Micro-Checker. Significant cases of heterozygote excess are offset to the left;

* loci exhibiting significantly poor fit to the hard sweep model (see Fig.3).

In a sexual mating system, a common cause of significant heterozygote excess at markers is locus (v) copy number variation. A large, 119S-specific CNV encompassing the region spanned by the outlying markers (i.e. ≥125 kb) could readily explain patterns of deviation from hard sweep predictions by inflating observed H_E_ in the 119S/S group. Unless there was actually more than one CNV (e.g. one encompassing locus −50 kb from G119S, and another encompassing loci at 29, 58 and 75 kb), it seems puzzling that the four microsatellites between −50 kb and 29 kb did not exhibit heterozygote excess. Null alleles in the 119S/S samples in three of these four loci, especially notable for locus ‘Ace-15k’, could mask this, but were not evident at Ace-5k, located within the *Ace-1* gene. To investigate this, we sequenced the Ace-5K locus directly from (uncloned) microsatellite PCRs in both 119G/G samples (all heterozygous at Ace-5k) and 119S/S samples (all homozygous at Ace-5k). As expected, the 119G/G individuals yielded sequence that deteriorated irretrievably from the heterozygous indel position in the microsatellite repeat region (Fig. S7, Supporting information). Surprisingly, each of the supposedly Ace-5k homozygous 119S/S individuals sequenced also showed evidence of indels within the repetitive region via a clear deterioration in sequence quality attributable to the presence of major and minor traces in the sequences (Figure S8, Supporting information). This suggests that scoring of Ace-5k 119S/S genotypes as homozygotes is erroneous, but arises because of a quantitative dominance of a single, presumably multiply copied allele, with the alternate allele in each heterozygote at a level of PCR amplification falling below our allele-scoring threshold.

We therefore conclude that the only explanation that can adequately and parsimoniously explain the deviation observed from the hard sweep model expectations and other population genetic parameters is the existence of a large CNV, detection of which was obscured at some loci by null alleles and/or presence of multiple, rather than duplicated copies.

### Copy number variation (CNV) of *Ace-1*

**Fig. 5:**
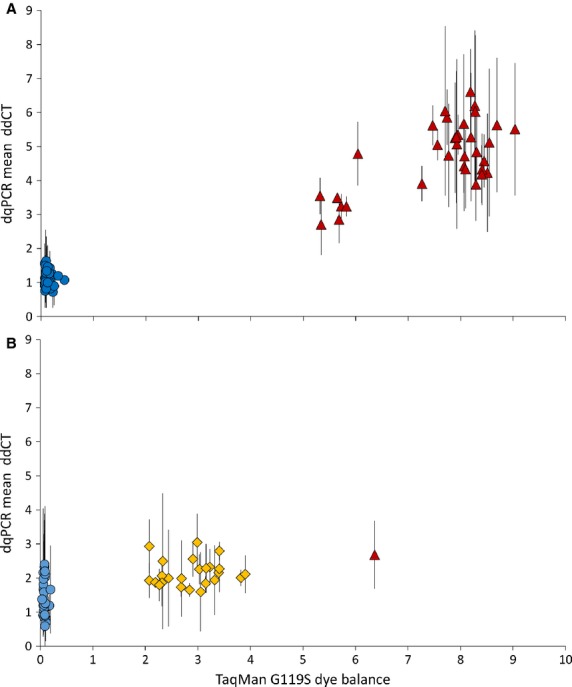
G119S polymorphism and *Ace-1* CNV estimation in *Anopheles gambiae*. (A) Samples from the 2008 Accra collection used for microsatellite genotyping and gene sequencing; (B) archived samples from several sites within Greater Accra sampled in 2002. Blue circle = 119G/G; red triangle = 119S/S; yellow diamond = 119G/S. The *x*-axis shows the dye balance ratio, which indicates the ratio of fluorescent signal from 119 serine (resistant): 119 glycine (susceptible) alleles at the qPCR endpoint. The *y*-axis shows *Ace-1* copy number estimated as ΔΔCT values calculated for products from three *Ace-1* exons (± standard deviation).

To test whether the evidence for segmental amplification above might correspond with CNV of *Ace-1*, we applied a recently developed qRT–PCR diagnostic (Edi *et al*. 2014) to the same sample used for microsatellite analysis. We detected a highly significant difference (heteroscedastic *t*-test, *t*_38_ = 20.4, *P* = 4.5 × 10^−22^) in estimated copy number between 119S/S (mean ± 95%CI = 4.75 ± 0.32) and 119G/G (mean = 1.12 ± 0.09) individuals (Fig.5A). In fact, there was no overlap between the sample groups with *Ace-1* in the vast majority of glycine homozygotes likely to be single copy (maximum estimated CNV ratio = 1.6) but all serine homozygotes clearly possessing multiple copies of resistant alleles (CNV ratio range 2.7–6.6). 119G/G genotypes cluster tightly, but 119S/S genotypes appear split into two main groups, especially with respect to the *x*-axis, raising the possibility that some of the TaqMan genotypes might have been mis-called (Fig.5A). Sequencing of the same *Ace-1* fragment as before in these samples confirmed the complete absence of 119G alleles; therefore, the split grouping appears to reflect differences in copy number.

We investigated the recent history of the *Ace-1* CNV by qRT–PCR of archived samples collected in 2002 from six sites in the Greater Accra district (all <100 km from the 2008 suburban Accra sample sites). Although only a few per cent of approximately *N* = 400, 2002 samples genotyped contained resistant alleles, *Ace-1* CNV was clearly present in 119G/S heterozygotes (Fig.5B); most (perhaps all) of these appear to possess additional alleles (range of CNV estimates 1.6–3.0). The single 119S homozygote in the 2002 sample set, which we confirmed as correctly genotyped by sequencing, exhibited a CNV metric within the range of heterozygotes. Again, there was a highly significant difference (homoscedastic *t*-test, *t*_52_ = 5.3, *P* = 2.2 × 10^−6^) in estimated copy number between 119G/S (mean ± 95%CI = 2.15 ± 0.16) and 119G/G (mean = 1.46 ± 0.19) individuals (Fig.5B). Variability in copy number in 119G homozygotes from 2002 was significantly greater than in 2008 (Levene's test, *F* = 5.1, *P* = 0.029), and the mean copy number estimate was significantly higher (*t*_52_ = 3.18, *P* = 0.003). Thus, in contrast to the 2008-collected 119G/G samples, at least some of the 2002 119G homozygotes were duplicants, consistent with the evolution of reduced CNV level of susceptible alleles over the relatively short time period between collection dates, whereas resistant alleles appear to have evolved towards increased copy number.

## Discussion

### Functional constraint and purifying selection acting on *Ace-1*

The G119S substitution in *Ace-1* is one of the most widespread insecticide resistance mutations known in mosquitoes (Weill *et al*. 2004a,b) and has evolved multiple times (Weill *et al*. 2004b). G119S polymorphism is strongly associated with resistance to carbamates and organophosphates (Djogbénou *et al*. 2007; Edi *et al*. 2012; Essandoh *et al*. 2013), with near-ubiquitous survival of homozygotes to standard WHO diagnostic doses in both *A. coluzzii* and *A. gambiae s.s*. (Essandoh *et al*. 2013). In comparison with the only other target site for WHO-approved insecticide classes, the *para* voltage-gated sodium channel (*Vgsc*), few resistance-associated mutations have been detected in *Ace-1* across insect species (IRAC 2014), suggesting marked functional constraint (Weill *et al*. 2003; Oakeshott *et al*. 2005; Alout & Weill 2008).

Sequencing results from our study strongly support this hypothesis, with significant statistical and qualitative evidence for purifying selection acting on wild-type 119G alleles. Despite very high synonymous nucleotide diversity, there were few amino acid variants; all (other than G119S) were very rare and with little predicted functional effect. These variants are probably transient variants in the normal birth and death process of mutations subject to small selection coefficients, with little chance of accumulation or increase in frequency in such a functionally constrained gene.

In marked contrast to the high 119G synonymous nucleotide diversity, but entirely consistent with purifying selection, the 119S mutation was the only variant of any kind detected in resistant haplotypes. Strong constraint on a protein poses a problem for the evolution of altered function, a highly desirable property for insecticide design, because coding variants of major structural impact are very likely to be deleterious. As discussed below, this is the case for the *Ace-1* 119S mutation, but unfortunately, *A. gambiae* appears to be in the process of circumventing this problem via gene amplification.

### Strength and nature of selection on *Ace-1* G119S

*A priori* expectations for the strength of directional selection operating on *Ace-1* were unclear. Despite very strong resistance association, AChE with the 119S amino acid substitution exhibits seriously impaired enzymatic activity in the absence of insecticide (Alout & Weill 2008). In *Culex pipiens*, this results in major fitness costs in field populations (Labbé *et al*. 2007b), which are also predicted in *A. gambiae* owing to similar interactions of AChE with insecticides in each species (Alout *et al*. 2008). Strong fitness costs have proved more difficult to detect in laboratory studies of *A. gambiae* (Djogbénou *et al*. 2010), but consistent with the cost hypothesis, 119S homozygotes are typically very rare or absent in wild populations, even where heterozygotes are extremely common (Djogbénou *et al*. 2008b; Ahoua Alou *et al*. 2010; Edi *et al*. 2012). An additional uncertainty arises because until very recently, carbamates and organophosphates played little part in organized vector control in West Africa, and accurate records of their scale of use in agriculture are unavailable. Neverthless, we unambiguously document genomic signatures of selection that are clearly centred on *Ace-1* G119S. Overall, our results suggest selection of 119S from a rare haplotypic background in a genomic region of high diversity. The area of the selective sweep spans at least 760 kb and, based on patterns of differentiation and extended LD, may exceed 2 Mb, though recombination appears likely to be impacting at least some of the loci beyond the 760-kb area. Whilst the span of the selective sweep area is symmetrical, both relative heterozygosity and differentiation show marked asymmetry about *Ace-1*. This occurred at least in part because of loci showing heterozygote excess, which significantly impacted those located from position −50 kb (from G119S) to 75 kb, and result primarily from possession of a pair of very common alleles as part of a duplicated genomic segment. Notably, sequencing of the microsatellite within the *Ace-1* intron detected evidence of CNV, but with one or more low-frequency additional alleles masked from genotype scoring by a single numerically dominant allele. Indeed, the magnitude of the signal of reduced diversity in the locale of *Ace-1* is clearly underestimated owing to the presence of the regional duplication.

In addition to the genomic signatures of selection, temporal variation in allele frequencies suggests that the 119S mutant is under substantial contemporary selection. The frequency of the 119S allele is increasing in Accra, which is at the centre of a localized belt of high-prevalence carbamate and organophophate resistance in Ghana (Essandoh *et al*. 2013). Whether public health use of insecticides has played any part in this selection is unclear, but a major role can probably be ruled out.

In 119S homozygotes, lack of variation in *Ace-1* and in microsatellites upstream (i.e. in the telomeric direction), and near maximal differentiation from 119G homozygotes, are entirely consistent with expectations of a hard selective sweep from a single very rare mutation (Messer & Petrov 2013). Similarly, LD in the 119S sample group was perfect within the sequenced region of *Ace-1* and generally elevated across the region screened by microsatellites, as would be predicted under a hard sweep. However, overlaid duplicated alleles, identical in *Ace-1* gene sequence, but not in flanking regions, clearly indicate incomplete identity by descent. The duplicated region appears large, at least 125 kb, and consequently extends far beyond the 11 kb *Ace-1* gene. Such a CNV size is similar to that detected recently in *Drosophila melanogaster* around the *Rdl* insecticide resistance target site locus (Remnant *et al*. 2013), though here, the gene itself is several times longer than *Anopheles Ace-1*. In spite of the large size of the CNV we detected, only three other genes are located therein (AGAP001357, 8 and 60), all downstream (i.e. centromeric direction) of *Ace-1*. Two are undescribed with no, or poor, orthology to functionally validated genes in other species, and the third has a good orthologue in *D. melanogaster*, but with an uncharacterized gene. None have any known or putative link to insecticide resistance and therefore seem unlikely to contribute directly to resistance phenotypes.

### *Ace-1* duplication and resistance evolution in mosquitoes

Gene duplication is thought to be a critical component of adaptive evolution (Ohta 1989; Ubeda *et al*. 2014) and probably remains an underestimated force in the evolution of insecticide resistance (Ffrench-Constant 2013). Copy number variation of *Ace-1* has been found in several diverse economically or medically important insect taxa and is often, though not always, linked to insecticide resistance (Labbé *et al*. 2007a; Djogbénou *et al*. 2008b; Kwon *et al*. 2010; Shang *et al*. 2014; Sonoda *et al*. 2014). Since its discovery in *A. gambiae s.s*. and *A. coluzzii* (Djogbénou *et al*. 2008a), the working model for the structure and function of *Ace-1* duplication has been based on *C. pipiens* mosquitoes (Alout *et al*. 2008). *C. pipiens* exhibit linked pairing of resistant and susceptible alleles on the same chromosome, which compensates for the fitness cost of possessing resistant alleles (Labbé *et al*. 2007a,b). This model seemed potentially applicable to *A. coluzzii* from southern Côte d'Ivoire that exhibit near fixed G119S heterozygosity and high *Ace-1* CNV frequency (Ahoua Alou *et al*. 2010; Edi *et al*. 2012, 2014). Similarly, in our archived 2002 Ghanaian samples (in which serine alleles were very rare), we found that, with the exception of one 119S homozygote, duplication involved G119S heterozygotes, all of which were duplicated. However, we also found a few 119G/G homozygotes duplicated at a low level. These were not found in the 2008 collection, all of which were single copy. This might indicate that, at least when the 119S mutation was very rare, duplication of susceptible alleles was favoured. In *Drosophila melanogaster* strains, low-level duplication of AChE provides enhanced insecticide resistance (Charpentier & Fournier 2001). This in turn suggests occurrence of transitional states in the evolution of resistance, involving replacement of duplicated susceptible alleles with linked point mutation and copy number variation. Such heterozygotes with an excess of duplicated resistant alleles may be either a stable alternative or a transitional state on the evolutionary path towards resistant homozygotes with many gene copies. Given the speed of change in allele and duplicate frequencies, this question may be resolved in the near future.

Possession of multiple copies of functionally identical *Ace-1*-resistant alleles by single insects does not appear to have been documented previously. The closest precedent to our results comes from two-spotted spider mites, *Tetranychus urticae*, which can possess a similarly high number of copies of resistant *Ace-1* alleles (Kwon *et al*. 2010), though with a mixture of three different resistance-associated substitutions (at different codons). Kwon *et al*. (2012) demonstrated that resistant alleles in *T. urticae* reduce the catalytic capacity of AChE, but that in the presence of these mutations, multiple copies of AChE restore catalytic activity to a normal (wild-type) level. This may provide a useful working model for multicopy 119S *Ace-1* in *A. gambiae*, though possession of up to six or copies of a region of at least 125 kb would suggest far from trivial costs in terms of significantly increased genome size that must be outweighed by adaptive value (Adler *et al*. 2014). Copy levels of susceptible alleles (i.e. beyond a simple qualitative CNV vs. no CNV distinction) did not provide enhanced resistance in *D. melanogaster*, and whether and how high level amplification might play a role in insecticide resistance is unclear. It is also currently unknown if the copies are arranged in tandem and thus how likely they are to be co-inherited, although preliminary cytological studies are consistent with this (I. Sharakov, L. S. Djogbenou, unpublished data). In spite of these uncertainties, given that *Ace-1* 119S/S homozygotes appear near-ubiquitously resistant to standard diagnostic doses of each class of insecticide (Essandoh *et al*. 2013), the strongly increasing frequency of 119S with a proportionate increase in homozygotes (G119S was in Hardy–Weinberg equilibrium in all collections) is an unexpected and extremely worrying trend.

### Detection of selective sweeps

A more positive implication of our study is that even in areas of apparently normal recombination in a very low LD genome (Harris *et al*. 2010; Neafsey *et al*. 2010; Weetman *et al*. 2010), which is likely to be characteristic of many medically and agriculturally important insect species, it is possible to detect very clear signatures associated with positive selection. The presence of a single *Ace-1* 119S sequence haplotype combined with the very high nucleotide diversity and very low LD in the *Ace-1* 119G wild-type haplotypes clearly argues for a recent origin by rare mutation rather than selection from standing genetic variation. However, as observed for drug resistance locus *pfmdr1* in *Plasmodium falciparum* (Nair *et al*. 2007), CNV causes deviation from hard sweep model expectations and reduces the local strength of the drop in diversity around the target gene. CNV identification is not currently a routine component of the selective sweep detection armamentarium for most organisms (but see (Nair *et al*. 2008)). Whilst automated identification of large CNVs remains challenging in genome scans, our results highlight that if CNVs are not detected, important signals of selection might be misinterpreted or missed entirely.

## Data accessibility

Microsatellite and *Ace-1* TaqMan genotyping data and qRT–PCT CNV analysis data are provided as supporting information in file: Weetman_et al. Data S1.xlsx. Sequence data have been submitted to GenBank with Accession nos.: KP165332–KP165384. Aligned sequences are also provided as supporting information in file: Weetman_et al. Data S2.phy.

## Supporting information

**Table S1** Details of microsatellites screened. **Table S2** Non-synonymous changes observed in *Ace-1* 119 wild-type haplotypes.

**Fig. S1** Recombination rate across chromosome 2R estimated from data in Zheng *et al*. 1996. **Fig. S2** Statistical parsimony analysis of 1266 bp of coding sequence surrounding codon 119 of the *Ace-1* locus. **Fig. S3** Estimation of the selection coefficient from deterministic model simulations. **Fig. S4** Allele frequency distributions at each microsatellite locus. **Fig. S5** As Fig. 3 but plotted for samples from a single location: the Accra suburb of Dzorwulu. **Fig. S6** Pairwise linkage disequilibrium (LD) heatmap between microsatellite loci. **Fig. S7** Sequencing of microsatellite Ace-5k in 119G/G individuals. **Fig. S8** Sequencing of microsatellite locus ‘Ace-5k’ in 119S/S individuals.

**Data S1** *Ace-1* 119 genotypes and microsatellite alleles for each locus in each individual genotyped and qRT–PCR CNV analysis results. File: Weetman *et al*. Data S1.xlsx

**Data S2** Alignment of *Ace-1* haplotypes in Phylip format. File: Weetman *et al*. Data S2.phy (text file).
