# Supplementary material for: Contemporary evolution of resistance at the major insecticide target site gene Ace-1 by mutation and copy number variation in the malaria mosquito Anopheles gambiae
Source: Mol Ecol. 2015 May 14;24(11):2656–72. doi: 10.1111/mec.13197 (PMC4447564; doi:10.1111/mec.13197)
Supplement: Supplementary file 1 [file mec0024-2656-sd1.pdf]

Table S1. Details of microsatellites screened

Locus name	G119S distance	AGAMP3 position	product	primer 1	primer 2	T_A/$MgCl_2$*
Ace-2.12M	-2117704	1374397	114	CGGTCTGGATTTTCAACTGG	CTTTGCAGTCTCGCCCTTT	55 +
Ace-1.03M	-1030997	2461104	177	CCAAGACGGACTTTTTCGAC	TGCAGGTATTGCTGTTGCTT	65
Ace-554K	-554790	2937311	126	CAAGGGGACGATGTGCTAAT	GTTGATTTCAGCTTGCGACA	65
Ace-372K	-372310	3119791	181	TTCAGTGGAACCGGAAAATC	GGCGTTGAATACGCTACCAT	55 +
Ace-322K	-322758	3169343	178	AGCTGTCGCTATGGTCGTTT	ACATATTGCGCACTCCATCA	55 +
Ace-199K	-199406	3292695	174	CGATTGCAAGCAGTAAGTCG	CCGGAACCATTTATCATCTCC	60
Ace-151K	-151330	3340760	166	GTTGATACAGCGTTGGCTTG	GTCGATCAAATGTTGGCTTC	58 +
Ace-103K	-103055	3389038	181	CAACCCCTCGTGTAAATTGG	TTCCCTCCAAAGTGTTTTGC	58 +
Ace-73K	-72500	3419595	285	ATGCTGGTGCGTGATAAATG	GAGGCATATCCTTCCTGTGC	58 +
Ace-50K	-50142	3441950	136	CATTCCACATGCTTCAAACG	CAATCCTTTTGGTCGTGCAG	62
Ace-24K	-24036	3468063	221	TGCCGTATTTCAATGTCAGC	ACAACCACCACCCTAACTGC	62
Ace-14K	-13867	3478223	116	ACCCAGTGGCTTGCTATTTG	TGCTCAATCGTTGTTGGAAG	60 +
Ace-5K	-5652	3486448	217	GTCCCTGCCCACACTCATAC	CTTCCAGCAAACCACAATCC	62
Ace+15K	+15348	3507439	130	TGGGATGGTTAGCCAGAAAG	TCCGTACCAAGATGATGTCC	58 +
Ace+29K	+28739	3520828	241	AAAAGGACTGTACCGCAACG	AATGGAAGTCGTCCCATCTG	60
Ace+58K	+57945	3550041	203	GAAAGGCACACAGACCAACC	TTTCACCACGAAACATCTGC	60 +
Ace+75K	+74735	3566843	254	GGTTGTAGTGCGGGGTTG	GCATTGAAACGCATTCGAC	65
*Ace+102K***	*+102069*	*3594167*	*252*	*TAATTCCCATTGCAAACGTC*	*CAGCTTGTGCCCTTCTTCTC*	*59*
Ace+150K	+149469	3641571	164	TCGCCTAGTTCTGTCGTTTG	CGAGTGTGGTTTTGAACCTG	58 +
Ace+193K	+192693	3684783	178	AGCCACTTGCCTGATAGTGC	AGCCAATCGTGAACAGGAAG	60 +
Ace+271K	+271479	3763580	222	TATGCTGCTGTGGCGTAATC	CAAAGAAAGCAAGTGTTTGGTG	62
Ace+388K	+388026	3880127	208	GGCGGAAATGAGAGTAAACG	TAGCCCGTAGTTCGTGTGTG	65
Ace+606K	+605602	4097703	130	TGACACATCGCAACTCCTTC	CGCGTGTAGTAAAGCCATCA	58
Ace+1.00M	+1000483	4492584	158	CGCCTCTAAGTCTTCGGTTG	GGAAACTGGGTTGCATTTGT	56 +
Ace+2.04M	+2042473	5534574	211	GTCATTGTGGCGGGATTTAC	GTTTGTGTCGTTGTGGTGGA	62

*Locus-specific annealing temperature (^{o}C), + indicates 0.5 µl of $MgCl_2$ added to PCR mixture; **excluded owing to unreliable scoring

Table S2. Nonsynonymous changes observed in *Ace-1* 119 wild-type haplotypes. Each change was observed in only one haplotype. Note that the numbering follows the *A. gambiae* AGAMP3 sequence rather than that of *Torpedo* enzyme used elsewhere (*Torpedo* codon 119 corresponds to *A. gambiae* codon 280). Of the seven that could be modelled none are predicted to radically affect gene function.

Amino acid in *A. gambiae* ACE-1	Common allele	Variant allele	Characterisation of position: solvent exposure in model and sequence conservation	Predicted effect on protein activity
115	Ala	Glu	Not in model, unconserved	*No structure-based prediction possible*
192	Leu	His	Semi-exposed, unconserved	His is not found in other species, but because other hydrophilic residues also are, it not predicted to have an impact.
262	Ala	Ser	Buried, unconserved	The larger amino acid Val is present in close relatives, so the small increase in size here unlikely to have an impact.
286	Ala	Thr	Semi-exposed, unconserved	Thr is found in some other species. Not predicted to have an impact.
323	Glu	Asp	Exposed, unconserved	Asp is present in some close relatives, so this conservative amino acid change is not predicted to have an impact
340	Val	Met	Buried, conserved as Ile, Val or Leu	Conservative change to Met; though slightly larger this is unlikely to have major consequences.
361	Ala	Thr	Buried, semi-conserved	Positioned close enough to both the catalytic Ser and to G280S (= *Torpedo* G119S) to possibly impact on catalytic activity or insecticide binding. However, Thr found in a few other species, e.g. *Bombyx mori*, arguing against a significant impact on catalytic activity.
477	Glu	Asp	Exposed, unconserved	Asp is present in some close relatives so this conservative change is not predicted to have any consequences.
