# Supplementary material for: Contemporary evolution of resistance at the major insecticide target site gene Ace-1 by mutation and copy number variation in the malaria mosquito Anopheles gambiae
Source: Mol Ecol. 2015 May 14;24(11):2656–72. doi: 10.1111/mec.13197 (PMC4447564; doi:10.1111/mec.13197)
Supplement: Supplementary file 2 [file mec0024-2656-sd2.pdf]

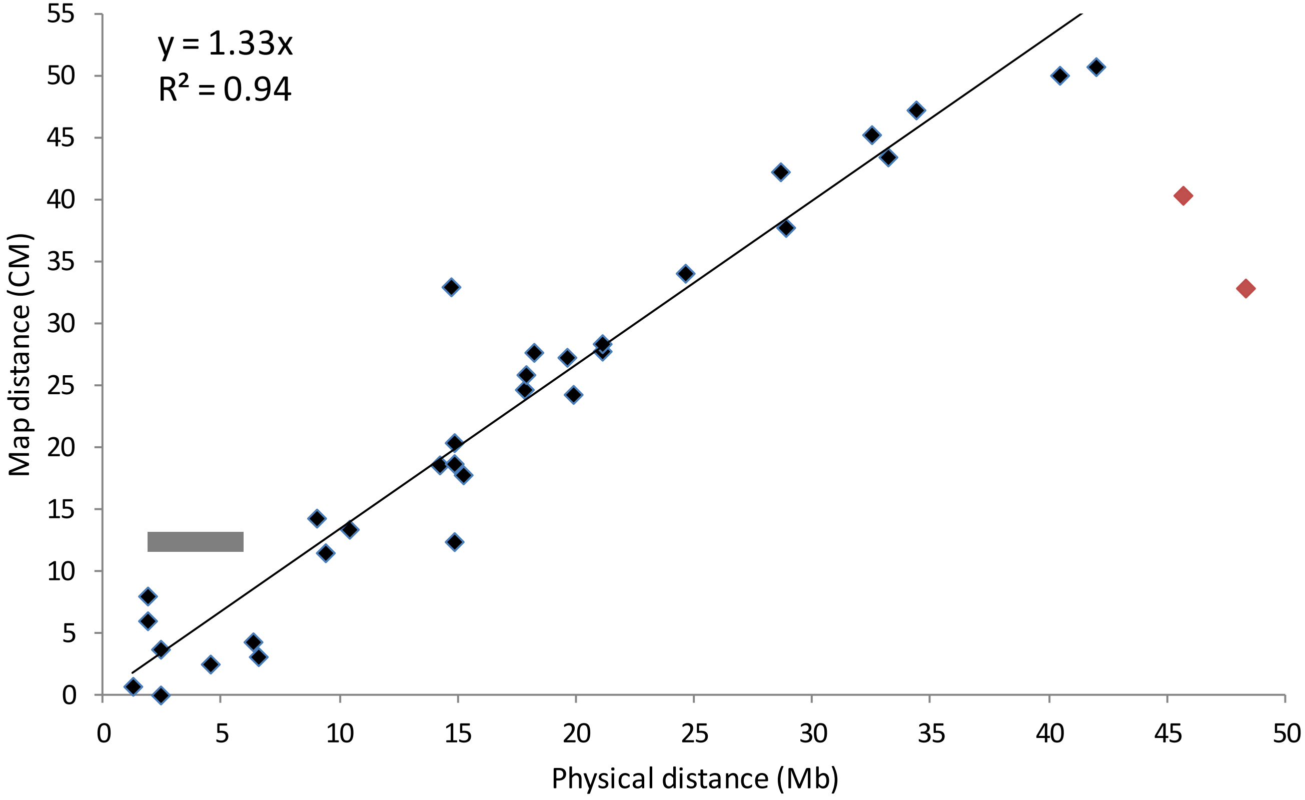

Fig. S1. Recombination rate across chromosome 2R estimated from data in Zheng et al. (1996). All 2R points are shown but those in red were excluded prior to fitting of the regression line; the slope provides the estimate of recombination rate (CM Mb^{-1}). The approximate area of the chromosome covered by the genotyping in the present study is indicated by the grey bar.

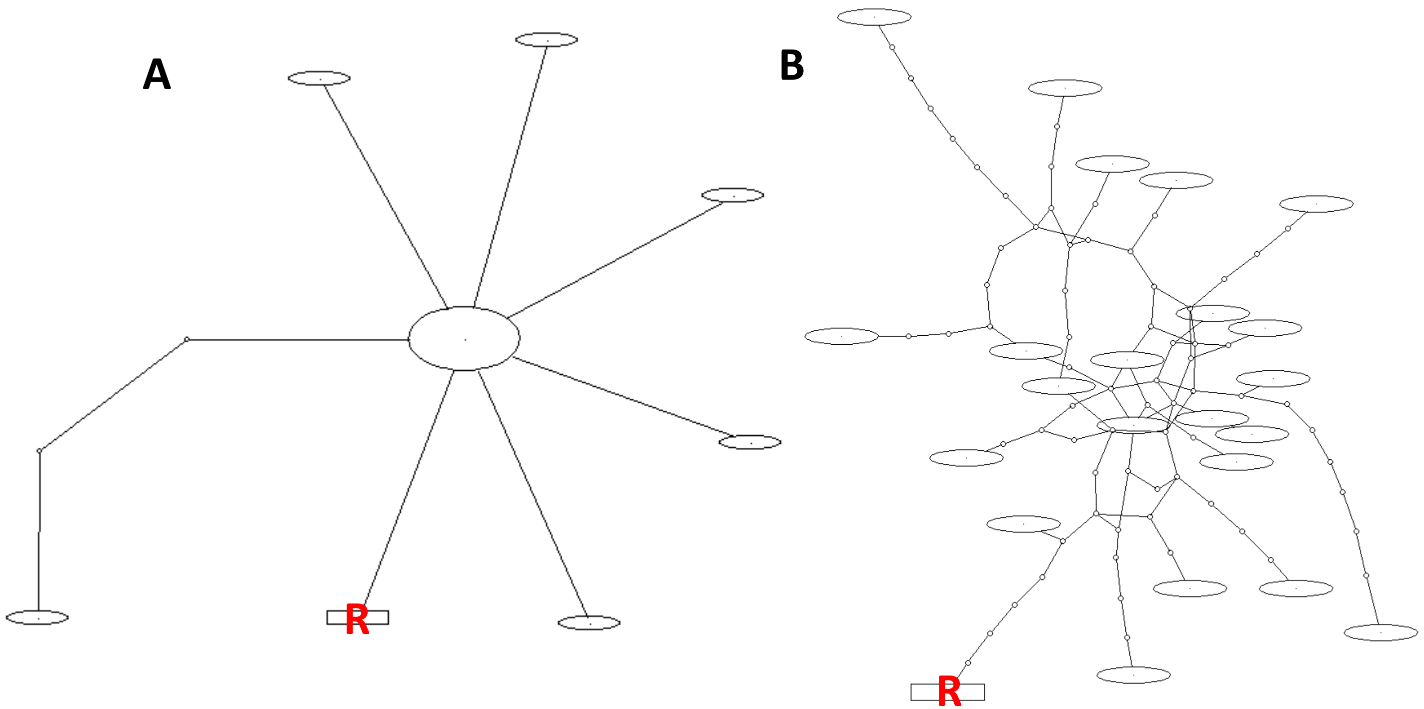

Fig. S2. Statistical parsimony analysis of 1266bp of coding sequence surrounding codon 119 of the *Ace-1* locus. Networks were produced from (A) non-synonymous and (B) synonymous mutations. In each plot small circles are unobserved haplotype nodes, ovals are 119G haplotypes and, except for the large oval in A (N=16), all are singletons. For information the rectangle in each plot shows the single resistant (**R**) haplotype found in all 119S/S individuals (not scaled to frequency). Note that in the non-synonymous plot the short branch lengths and star-like nature of the network - a common characteristic of (i) purifying selection or (ii) population expansion (Posada & Crandall 2001). The contrast with the long-branched, heavily-reticulated synonymous network supports (ii).

Reference: Posada D, Crandall KA (2001) Intraspecific gene genealogies: trees grafting into networks. *Trends in Ecology & Evolution*, **16**, 37–45.

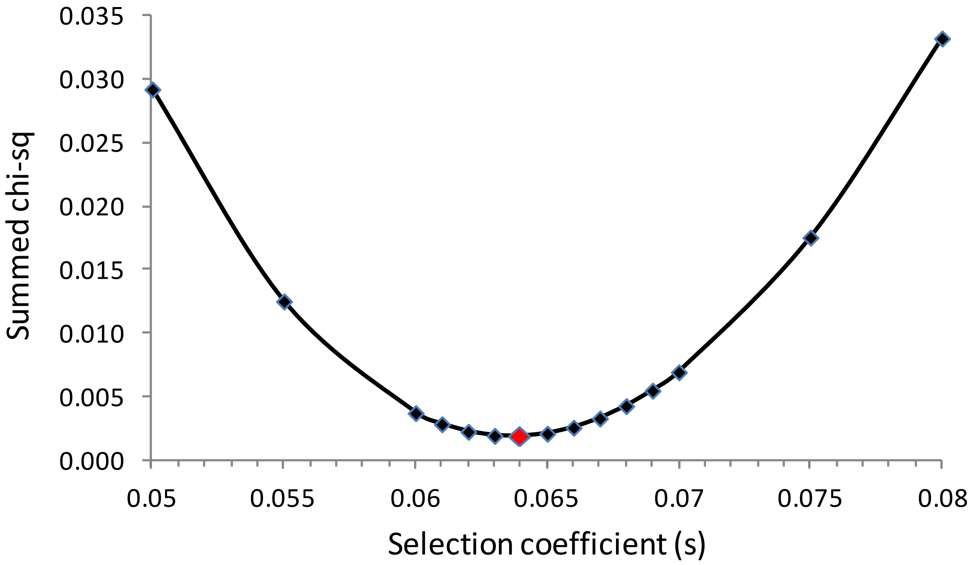

Fig. S3. Estimation of the selection coefficient (*s*) from a deterministic codominant model simulated using the software POPULUS and assessed using chi-square goodness of fit. Points show simulations; the highlighted red point is the best fit (*s*=0.064). The initial allele frequency was set from the intercept of the regression fit to observed data shown in Fig. 2.

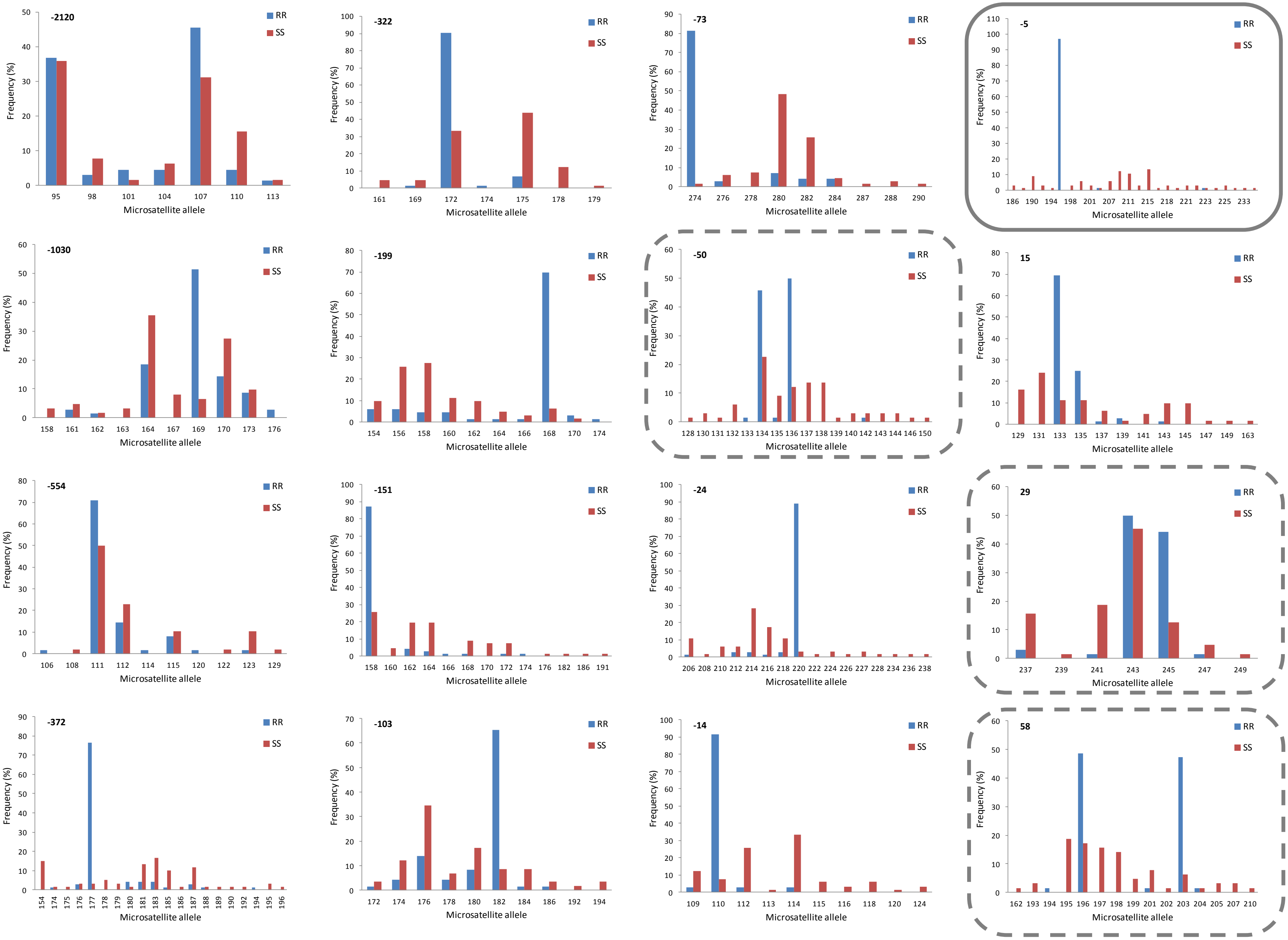
-2120
RR
SS
Frequency (%)
95 98 101 104 107 110 113
Microsatellite allele
-322
RR
SS
Frequency (%)
161 169 172 174 175 178 179
Microsatellite allele
-73
RR
SS
Frequency (%)
274 276 278 280 282 284 287 288 290
Microsatellite allele
-5
RR
SS
Frequency (%)
186 190 194 198 201 207 211 215 218 221 223 225 233
Microsatellite allele
-1030
RR
SS
Frequency (%)
158 161 162 163 164 167 169 170 173 176
Microsatellite allele
-199
RR
SS
Frequency (%)
154 156 158 160 162 164 166 168 170 174
Microsatellite allele
-50
RR
SS
Frequency (%)
128 130 131 132 133 134 135 136 137 138 139 140 142 143 144 146 150
Microsatellite allele
15
RR
SS
Frequency (%)
129 131 133 135 137 139 141 143 145 147 149 163
Microsatellite allele
-554
RR
SS
Frequency (%)
106 108 111 112 114 115 120 122 123 129
Microsatellite allele
-151
RR
SS
Frequency (%)
158 160 162 164 166 168 170 172 174 176 182 186 191
Microsatellite allele
-24
RR
SS
Frequency (%)
206 208 210 212 214 216 218 220 222 224 226 227 228 234 236 238
Microsatellite allele
29
RR
SS
Frequency (%)
237 239 241 243 245 247 249
Microsatellite allele
-372
RR
SS
Frequency (%)
154 174 175 176 177 178 179 180 181 183 185 186 187 188 189 190 192 194 195 196
Microsatellite allele
-103
RR
SS
Frequency (%)
172 174 176 178 180 182 184 186 192 194
Microsatellite allele
-14
RR
SS
Frequency (%)
109 110 112 113 114 115 116 118 120 124
Microsatellite allele
58
RR
SS
Frequency (%)
162 193 194 195 196 197 198 199 201 202 203 204 205 207 210
Microsatellite allele

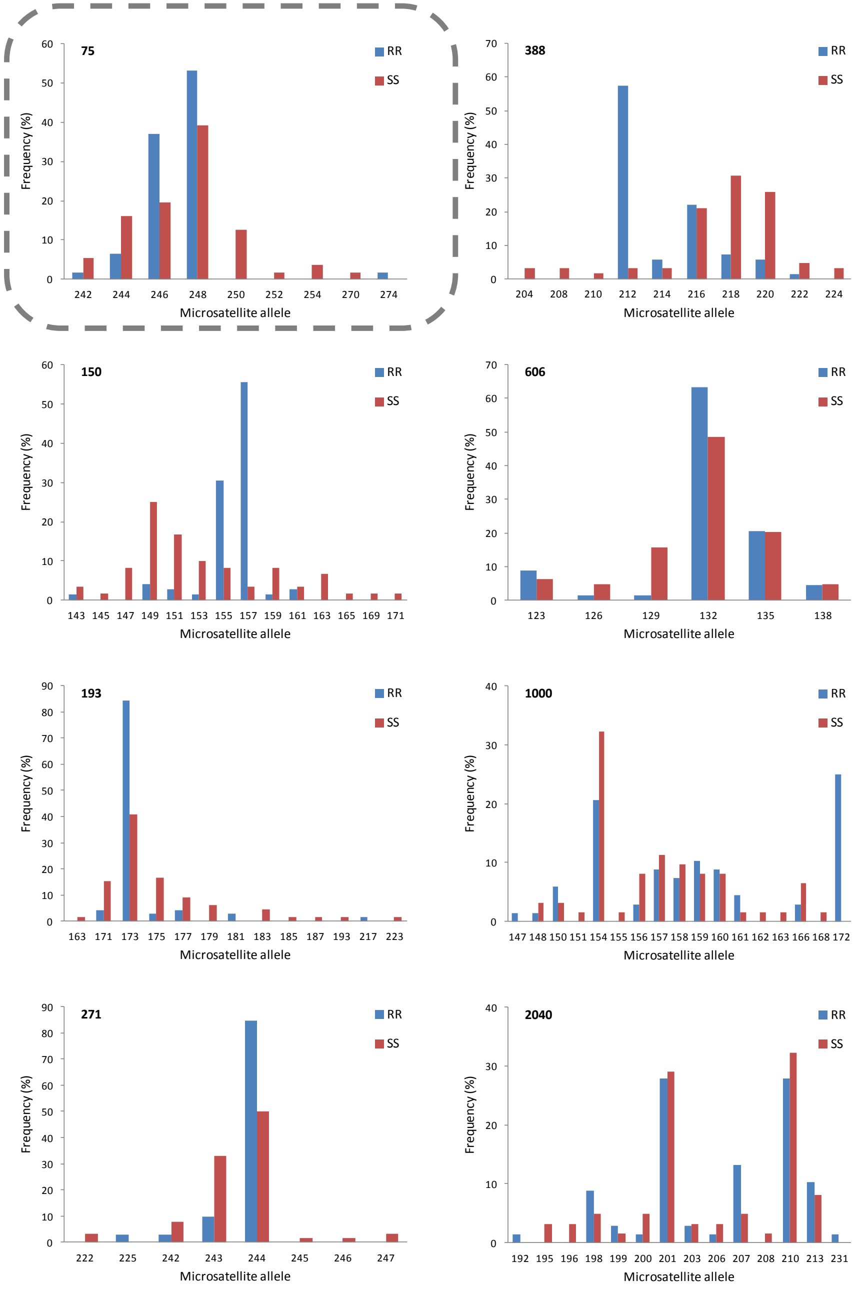

Fig. S4 Allele frequency distributions at each microsatellite locus (labelled according to distance from *Ace-1* G119S position) in the serine (resistant) and glycine (susceptible) homozygote groups (named RR and SS, respectively). The plot for the nearest microsatellite to *Ace-1* G119S, which shows a particularly extreme difference in profile between 119G/G and 119S/S, is outlined, and the plots for loci exhibiting significantly poorer hard sweep model fit are outlined in dashed boxes.

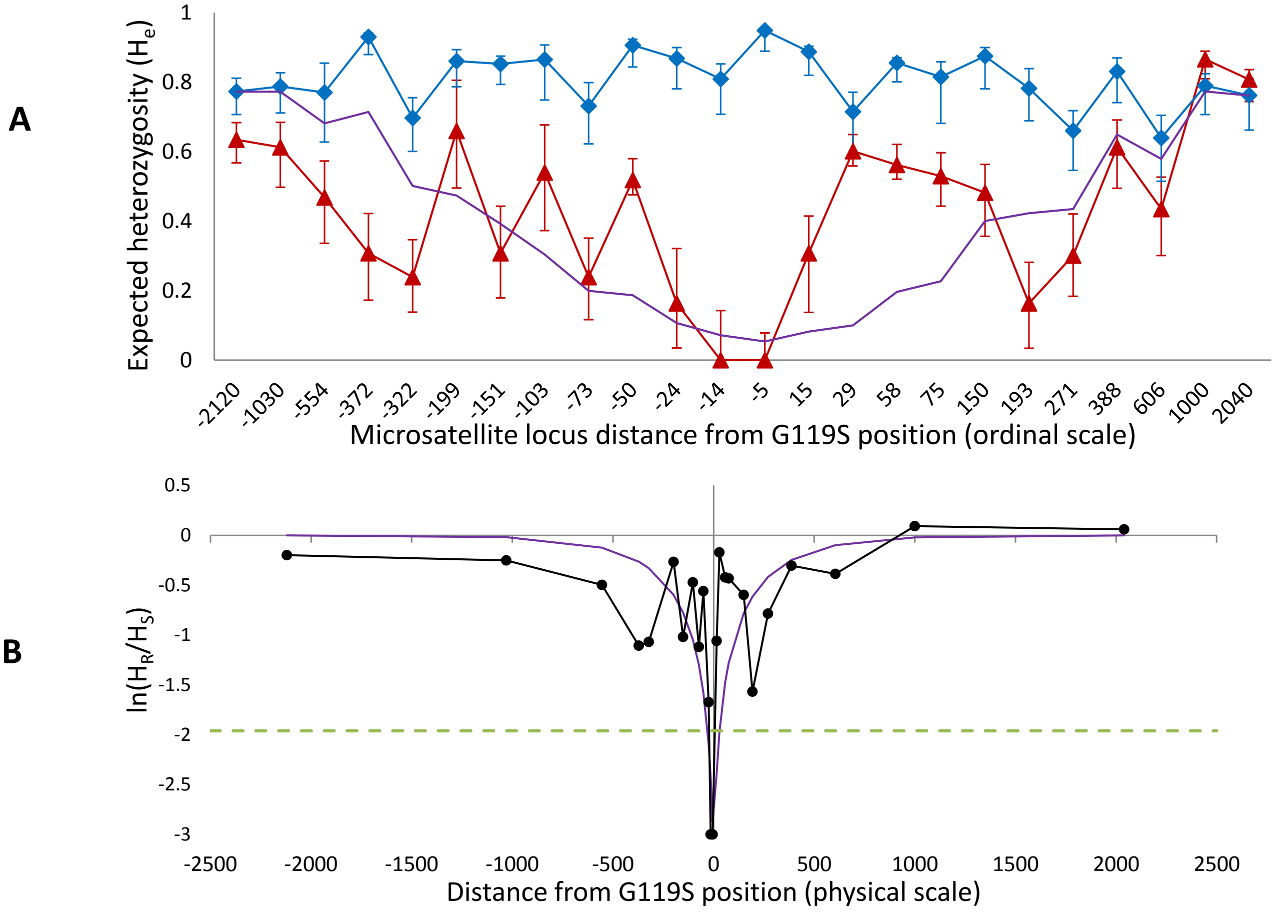

Fig. S5 As Fig. 3 but plotted for samples from a single location: the Accra suburb of Dzorwulu. (**A**) Heterozygosity ±95% CI for 119S (resistant) homozygotes (red triangles) and 119G (wild-type) homozygotes (blue diamonds); the purple line is a deterministic model prediction for a hard selective sweep derived from the wild-type heterozygosities, employing realistic parameter estimates for the selection coefficient, mutation and recombination rates. (**B**) Heterozygosity ratio $\ln(H_R/H_S)$ with the dashed line showing the 95% significance threshold for a two-tailed Z-test; the purple line shows the equivalent model prediction to that in A. Note that in B the microsatellites closest to 119S showed zero heterozygosity so $\ln(H_R/H_S)$ values are set to -3.

SS / GG	-2120	-1030	-554	-372	-322	-199	-151	-103	-73	-50	-24	-14	-5	15	29	58	75	150	193	271	388	606	1000	2040
-2120	T	0.21	0.83	1.00	0.59	0.03	0.18	0.90	0.32	1.00	0.73	0.34	1.00	0.91	0.11	1.00	0.03	0.09	0.19	0.72	0.13	0.66	0.60	0.40
-1030	0.08		0.84	1.00	0.77	1.00	1.00	0.03	0.85	1.00	0.90	0.94	0.47	0.79	0.47	0.65	0.47	0.81	0.58	0.20	0.05	0.93	0.77	0.22
-554	0.78	0.35		0.38	0.43	0.79	0.67	0.01	0.16	0.46	0.82	0.25	0.07	0.83	0.61	0.46	0.91	1.00	0.83	0.52	0.38	0.99	0.65	0.53
-372	0.38	0.01	0.04		1.00	0.20	1.00	1.00	1.00	1.00	0.33	1.00	1.00	**0.00**	1.00	1.00	0.17	1.00	0.40	0.47	1.00	0.40	1.00	0.19
-322	0.50	0.87	0.51	0.00		0.32	0.94	0.22	0.04	0.58	0.45	0.29	0.32	0.95	0.67	0.82	0.23	0.33	0.83	0.92	0.05	0.67	1.00	0.43
-199	0.08	0.70	0.57	0.05	0.01		0.76	0.17	0.38	0.20	0.73	0.74	1.00	0.40	0.14	0.56	0.12	0.23	0.99	0.70	0.98	0.67	0.05	0.03
-151	0.40	0.21	0.20	0.01	0.07	0.19		1.00	0.95	1.00	1.00	0.57	1.00	0.09	0.84	0.10	0.31	0.50	0.49	0.79	1.00	0.06	0.38	1.00
-103	0.07	0.12	0.07	**0.00**	0.66	0.01	0.14		0.04	0.36	0.78	0.42	0.36	0.72	0.12	0.50	0.29	0.46	0.61	0.12	0.15	0.93	1.00	0.12
-73	0.02	0.12	0.00	**0.00**	0.13	0.00	0.01	**0.00**		1.00	0.86	0.64	0.59	0.61	0.17	0.48	0.79	0.27	0.88	0.98	0.06	1.00	0.84	0.85
-50	0.86	0.07	0.29	0.03	1.00	0.77	0.59	0.11	0.29		1.00	1.00	0.21	1.00	0.68	1.00	1.00	0.19	1.00	0.72	1.00	1.00	1.00	0.01
-24	0.07	0.17	0.00	0.04	0.77	0.36	0.03	0.04	**0.00**	0.03		0.53	1.00	1.00	0.27	1.00	0.02	0.19	0.10	0.29	1.00	0.01	1.00	0.25
-14	0.62	1.00	0.36	0.52	0.62	0.75	0.19	0.68	0.32	1.00	0.53		0.59	0.77	0.48	0.29	0.35	0.89	0.86	0.36	0.54	0.42	0.40	1.00
-5	1.00	0.38	0.07	0.12	1.00	0.77	0.40	0.37	0.11	1.00	1.00	0.21	★	1.00	0.74	1.00	1.00	1.00	1.00	0.43	0.04	1.00	0.36	1.00
15	0.65	0.69	0.00	0.15	0.75	0.75	0.04	**0.00**	0.01	0.16	0.01	0.52	0.62		0.86	1.00	1.00	1.00	0.41	0.63	1.00	0.33	0.35	0.71
29	0.77	0.11	0.38	0.84	0.02	0.00	0.30	0.95	0.47	1.00	0.67	0.50	1.00	0.17		1.00	0.53	**0.00**	0.98	0.54	0.15	0.12	0.83	0.95
58	1.00	0.29	0.37	1.00	1.00	0.43	1.00	0.69	1.00	1.00	1.00	1.00	1.00	0.31	0.20		1.00	1.00	1.00	1.00	1.00	0.88	1.00	0.48
75	0.07	0.11	0.68	0.17	0.79	0.40	0.53	0.14	0.56	0.16	0.05	0.58	1.00	0.73	0.94	0.33		0.76	0.10	0.07	0.98	0.16	1.00	0.16
150	0.65	0.35	0.06	0.02	0.42	0.60	0.01	0.05	0.01	0.01	0.01	0.45	0.76	**0.00**	0.31	0.35	0.03		1.00	0.48	0.16	0.28	1.00	1.00
193	0.06	0.34	0.28	0.30	0.69	0.64	0.01	0.49	0.04	0.72	0.02	0.08	1.00	0.14	0.51	1.00	0.50	**0.00**		0.48	0.85	0.54	0.78	0.27
271	0.21	0.47	0.77	0.37	0.56	0.82	0.16	0.87	0.71	0.68	0.26	0.02	1.00	0.64	0.72	0.08	0.10	0.08	**0.00**		0.63	0.54	0.08	0.26
388	0.01	0.20	0.74	0.32	0.34	0.08	0.10	0.00	0.02	0.34	0.08	0.01	0.62	0.00	0.37	0.36	0.68	**0.00**	0.00	0.08		0.77	0.63	0.06
606	0.03	0.34	0.24	0.16	0.83	0.69	0.13	0.22	0.19	0.92	0.36	0.09	0.60	0.21	0.89	0.84	0.01	0.11	0.01	0.01	0.47		0.92	0.47
1000	0.21	0.38	0.24	0.25	0.90	0.95	0.22	0.34	0.12	0.05	0.07	0.21	0.81	0.19	0.70	0.62	0.30	0.41	0.08	0.01	0.08	0.02		0.54
2040	0.10	0.18	0.49	0.13	0.67	0.09	0.21	0.24	0.05	0.44	0.61	0.31	1.00	0.25	0.41	0.51	0.90	0.00	**0.00**	**0.00**	0.02	0.12	0.01	C

Fig. S6. Pairwise linkage disequibrium (LD) heatmap between microsatellite loci arranged on each axis in an ordinal scale with numbers showing distance in kb from *Ace-1* G119S (star). Values show probabilities (of no LD) for the wild type 119G/G sample group (above diagonal) and 119S/S sample group (below diagonal). The colour scale runs (high-low LD) red-orange-yellow-green. Cells with borders highlight values below P=0.05; values significant after multiple correction are shown in bold type. T and C indicate telomeric and centromeric orientations, respectively. LD is higher among microsatellites in 119S/S individuals (Wilcoxon test, z=7.94, P<0.0001), and 21% of all pairwise comparisons significant (at P<0.05), compared to 6% in 119G/G.

A

B

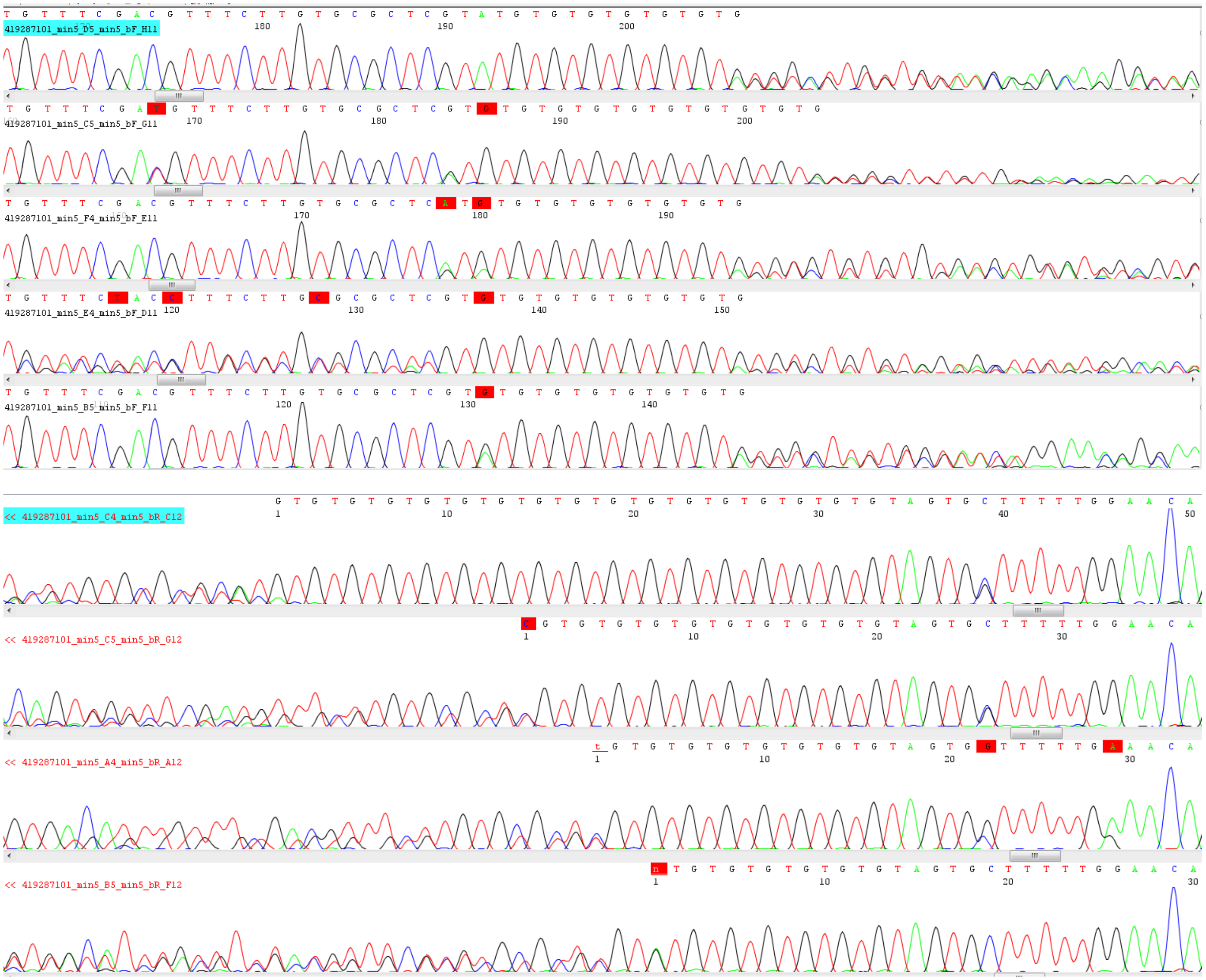

Fig. S7. Sequencing of microsatellite Ace-5k in 119G/G individuals. Trace files show the (TG) microsatellite sequence in each direction (**A**,**B**) for the same individuals (in the same order). Sequence is no longer readable after the repetitive region owing to the approximately equal strength traces, typical of heterozygous indels in directly sequenced PCR products.

A

B

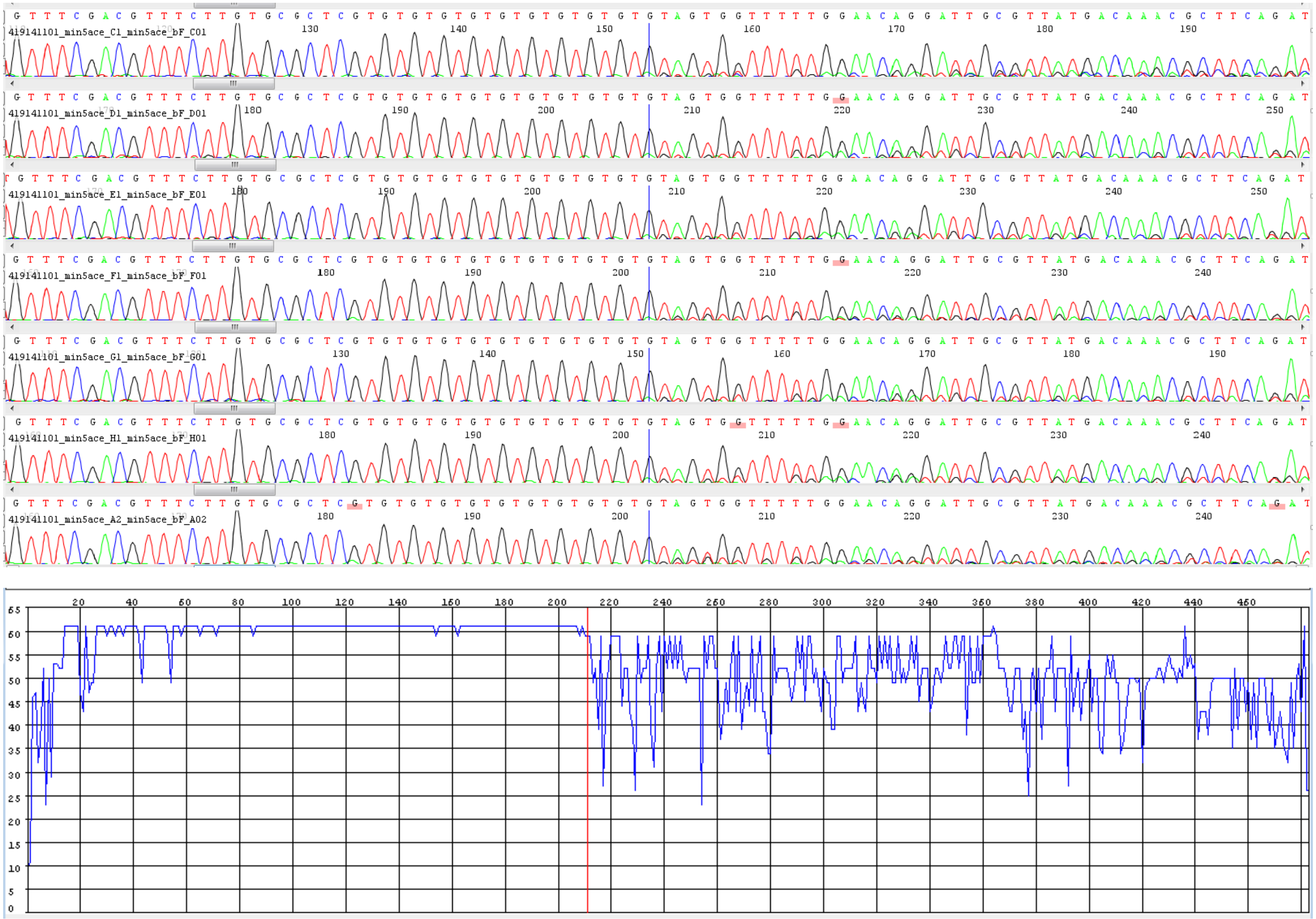

Fig. S8. Sequencing of microsatellite locus ‘Ace-5k’ in 119S/S individuals. (**A**) Trace files show the (TG) microsatellite sequence, which ends at the vertical line. Note that sequence is still readable thereafter but low amplitude traces are visible under each peak, reflected in the reduced PHRED quality scores, shown in (**B**); vertical line position corresponds to that in (A).
